# New antifungal strategies and drug development against WHO critical priority fungal pathogens

**DOI:** 10.3389/fcimb.2025.1662442

**Published:** 2025-09-25

**Authors:** Yanjian Li, Yang Liu, Yicong Jiang, Yusen Yang, Wanxing Ni, Wanli Zhang, Lingchen Tan

**Affiliations:** ^1^ College of Life and Health Sciences, Northeastern University, Shenyang, China; ^2^ School of Life Science and Bio-pharmaceutics, Shenyang Pharmaceutical University, Shenyang, China

**Keywords:** fungal infections, antifungal resistance, antifungal agents, drug development, drug design

## Abstract

Fungal infections pose a significant threat to human health, particularly in immunocompromised individuals, driving a sustained increase in the demand for effective antifungal agents. These agents can be classified into several categories based on their mechanisms of action and chemical structures, including inhibitors of sterol synthesis, cell wall synthesis, DNA synthesis, and cell membrane function. Each class exerts its antifungal effects through distinct molecular pathways that disrupt fungal cell growth and reproduction. However, the clinical utility of current antifungal therapies is hindered by challenges such as the emergence of drug resistance, limited antifungal spectra, and adverse side effects. Consequently, the development of safe and efficacious antifungal agents remains a pressing need. This review provides a comprehensive overview of the classification and molecular mechanisms of antifungal drugs, discusses the current challenges in antifungal therapy, and explores potential strategies for future drug development, aiming to inform and advance antifungal research and treatment.

## 1 Introduction

The incidence of fungal infections has significantly increased in recent years, ranging from mild allergic reactions to potentially life-threatening invasive fungal diseases (IFDs) (Kronstad et al., 2011; Stop neglecting fungi, 2017; Almeida et al., 2019; Fisher and Denning, 2023; Denning, 2024). Globally, over one billion people worldwide are affected by fungal infections each year, among whom more than 6.55 million suffer from fungal diseases that threaten their lives immediately (Denning, 2024). Such infections not only seriously damage the quality of life of patients, but also impose a heavy burden on the global healthcare system (Bongomin et al., 2017; Denning, 2024; Iliev et al., 2024).

**Figure 1:**
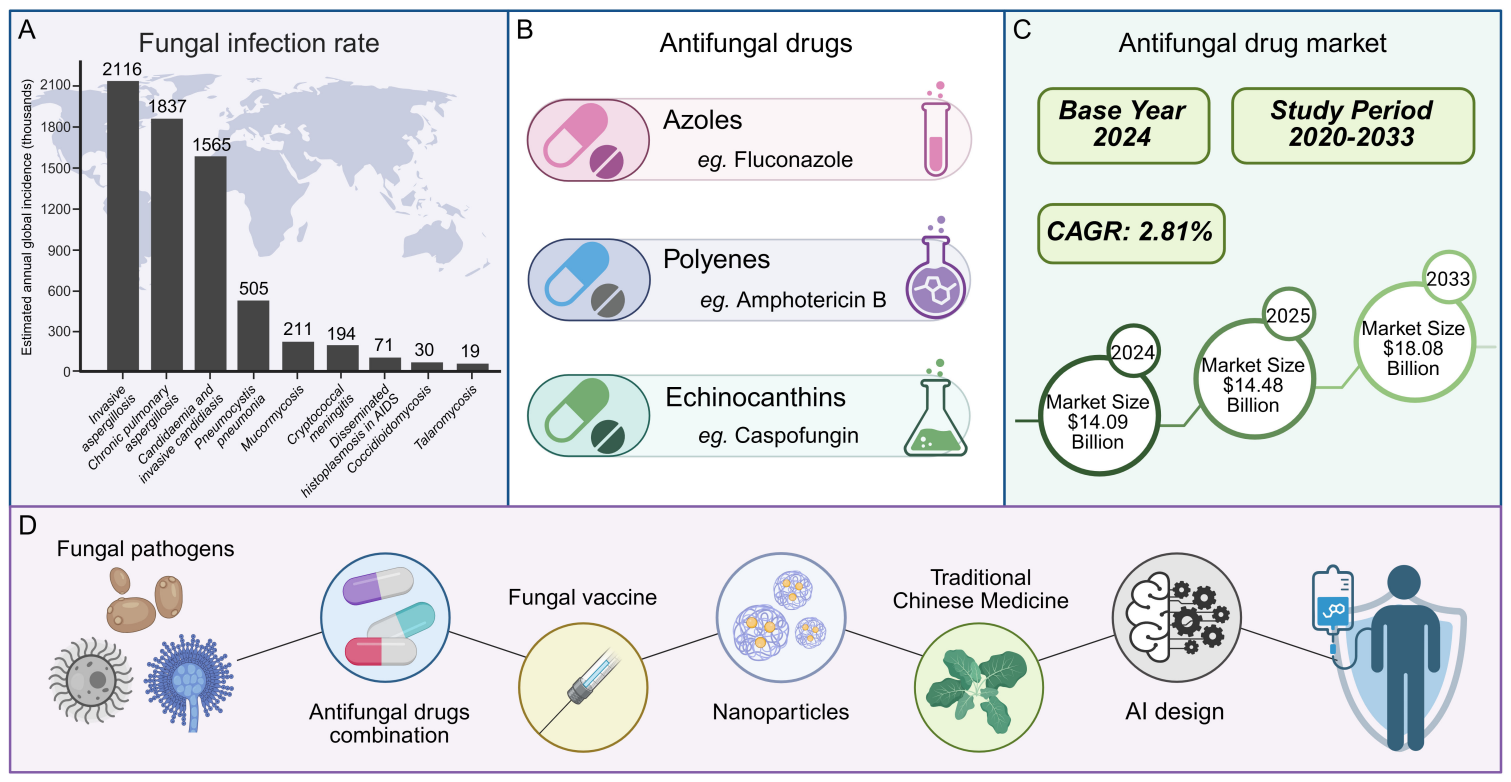
Global fungal infection challenges and antifungal efforts.

In the battle against human pathogenic fungi, antifungal drugs have emerged as indispensable tools. Currently, the treatment of invasive fungal infections relies exclusively on three main classes of antifungal drugs: polyenes, azoles, and echinocandins (Perfect, 2017). Consequently, the development of resistance by pathogenic fungi to any one of these drug classes would drastically narrow the range of available clinical treatment options. From a market perspective, the demand for antifungal drugs continues to rise: The market is expected to expand at a compound annual growth rate (CAGR) of 2.81% from 2020 to 2033, with a size of 14.09 billion US dollars in 2024, expected to rise to 14.48 billion US dollars in 2025, and reach 18.08 billion US dollars by 2033 (**Figure 1**) (Insights, 2025).However, the development and application of antifungal drugs face numerous challenges (Fisher et al., 2018; Iyer et al., 2021). Notably, with the increasing clinical utilization of antifungal drugs, the problem of fungal drug resistance has become increasingly prominent (Fisher et al., 2018; Vitiello et al., 2023; Iliev et al., 2024). The resistance rates of some fungi to existing antifungal drugs have risen significantly, directly resulting in treatment failures (Fisher et al., 2022; Antimicrobial resistance: a silent pandemic, 2024). Moreover, current drugs may suffer from inadequate selectivity for specific fungal species and significant side effects, which limits their widespread clinical use (Puumala et al., 2024).

With advancements in molecular biology and medicinal chemistry, research on antifungal drugs has been continuously progressing. New strategies for the development of antifungal drugs have gradually become a research hotspot, including the targeting of novel biological pathways, the combination of antifungal drugs, the development of fungal vaccines, the creation of innovative therapeutics (such as small-molecule peptides and nanoparticles), the extraction of active ingredients from traditional Chinese medicine, and the utilization of artificial intelligence in drug design (**Figure 1**) (Wall and Lopez-Ribot, 2020; Wu et al., 2023; Zhu et al., 2023; Saini et al., 2025). These studies offer new insights for the future development of antifungal medications. This review aims to systematically discuss the classification, molecular mechanisms, and drug development strategies in fungal treatment, providing valuable references for researchers and clinicians in related fields. Additionally, we will explore the current challenges in antifungal drug research and future directions, with the goal of promoting innovation and progress in the field of antifungal therapy.

This infographic outlines the landscape of fungal infections and antifungal efforts. Globally, fungal infections affect 6.55 million yearly, with rates climbing, as shown by the varied incidence of different fungal diseases (A, data are derived from *Denning, D. W., Lancet Infect Dis, 2024 (* Denning, 2024)). Antifungal drugs exist in classes like azoles, polyenes, and echinocandins, yet drug resistance complicates treatment (B). The market for these drugs, growing at a 2.81% CAGR from 2020–2033, was $14.09 billion in 2024, projected to reach $14.48 billion in 2025 and $18.08 billion by 2033 (C). To tackle challenges, new approaches are underway: combining antifungal drugs, developing fungal vaccines, creating innovative medications (including small - molecule peptides and nanoparticles), extracting actives from traditional Chinese medicine, and leveraging AI for drug design—all aiming to improve fungal infection management (D).

## 2 Major invasive fungal pathogens and their drug resistance status

Fungi represent a major biological kingdom with an extensive evolutionary history, comprising an estimated global diversity of over 5 million species, among which more than one million have been formally identified (Schueffler and Anke, 2014; Hawksworth and Lücking, 2017). While many fungi play beneficial roles in medicine (e.g., *Ganoderma lucidum*, *Poria cocos*), agriculture (e.g., mycorrhizal fungi enhancing plant nutrient uptake), and the food industry, a considerable number also pose serious threats to human health, agricultural productivity, and ecosystem stability. Notably, fungi have become one of the most formidable targets in anti-infective therapy (Lange, 2014; Liu et al., 2024; Cargill et al., 2025; Guo et al., 2025; Lei et al., 2025; Moora et al., 2025).

According to the World Health Organization (WHO), approximately 6.5 million cases of invasive fungal infections occur globally each year, resulting in an estimated 3.8 million deaths, of which 68% (around 2.5 million) are directly attributed to fungal diseases (Organization, W. H., 2022; Lockhart et al., 2023; Denning, 2024). Despite this, the threat of fungal pathogens to human health has long been underestimated. Regardless of economic development status, the incidence of invasive fungal diseases is steadily increasing, exerting a profound impact on public health (Seagle et al., 2021; Denning, 2024; Thompson et al., 2024a). Since 2016, *Aspergillus fumigatus* (associated with a mortality rate of 50%–90%), *Cryptococcus neoformans* (20%–70%), and *Candida albicans* (20%–40%) have been recognized as the leading causes of life-threatening fungal infections (Vandeputte et al., 2012; Han et al., 2016). In recognition of their clinical significance and growing resistance profiles, the WHO has designated these pathogens, along with *Candida auris*, as part of the “Critical Priority Group” in its Fungal Priority Pathogens List (Lockhart et al., 2023). Additionally, a growing number of emerging and re-emerging fungal pathogens, such as species of *Fusarium*, *Mucorales*, *Histoplasma capsulatum*, and *Sporothrix*, pose significant threats to immunocompromised populations. Although these pathogens are less prevalent globally, their morbidity and mortality may be substantially underestimated due to limited surveillance, low prioritization in public health frameworks, and geographically restricted endemicity. These pathogens often exhibit intrinsic or acquired resistance to antifungal agents, severely limiting treatment options. For example, *Fusarium solani* and *F. oxysporum* are intrinsically resistant to multiple antifungals, including azoles and echinocandins, making infections exceedingly difficult to treat (Dignani and Anaissie, 2004; Lortholary et al., 2010; Wiederhold et al., 2010; Demonchy et al., 2024). Although mucormycosis is rare globally, its incidence in India is approximately 80 times higher than elsewhere (Prakash and Chakrabarti, 2019; Skiada et al., 2020; Kottarathil et al., 2023). *Histoplasma capsulatum* is endemic to tropical and subtropical regions of the Americas (Teixeira Mde et al., 2016; Woods, 2016; Araúz and Papineni, 2021), while *Sporothrix* species (e.g., *S. schenckii*, *S. brasiliensis*, *S. globosa*) are closely linked to zoonotic outbreaks, particularly in China and Brazil (Alvarez et al., 2022; Rodrigues et al., 2022). The resistance profiles and regionally concentrated disease burdens of these fungi underscore the need for intensified mycological research.

It is noteworthy that fungal pathogens affecting plants and wildlife also pose significant global threats and offer unique insights into human fungal diseases. For instance, *Magnaporthe oryzae* (rice blast disease) and *Fusarium oxysporum* (vascular wilt in crops) are major plant pathogens that threaten global food security and cause substantial economic losses (Nalley L et al., 2016; Dita M et al., 2018). In the wildlife domain, *Batrachochytrium dendrobatidis* and *B. salamandrivorans* have been implicated in catastrophic amphibian population declines worldwide, while *Pseudogymnoascus destructans* is the causative agent of white-nose syndrome, which has led to mass mortality in North American bat populations (Drees KP et al., 2017; Grogan LF et al., 2018). Studies on these non-human pathogenic fungi have shed light on cross-species virulence mechanisms and provided valuable models for understanding fungal pathogenesis, host interaction, and immune evasion in humans. However, in the face of the rising burden of fungal infections, current treatment strategies remain inadequate.

Despite recent progress in antifungal therapy and the advancement of several novel agents into clinical trials, the rapid emergence of antifungal resistance has emerged as a critical barrier to effective treatment and is now recognized by the World Health Organization as one of the top ten global public health threats (Ferdinand et al., 2023; Vitiello et al., 2023). Currently available antifungal drugs are frequently associated with significant toxicity, pronounced side effects, narrow spectra of activity, and a high propensity for inducing resistance (Albrecht, 2019; Szymański et al., 2022; Zhou et al., 2022; Cavassin et al., 2024; Puumala et al., 2024; Gu et al., 2025). These limitations are particularly critical in the management of multidrug-resistant (MDR) and pan-drug-resistant (PDR) fungal infections, where therapeutic options remain extremely limited. These growing challenges underscore the urgent need for the development of antifungal agents with novel mechanisms of action, improved specificity, and reduced toxicity, alongside the identification of new molecular targets and therapeutic strategies to mitigate the escalating global burden of fungal diseases.

### 2.1 Candida

*Candida* species are major fungal pathogens, particularly in immunocompromised individuals, where infections are associated with high mortality, up to 45% despite antifungal therapy (Chowdhary et al., 2023; Robbins and Cowen, 2025). With the widespread use of antifungal agents, resistance among *Candida* species has become increasingly concerning, especially with the emergence of multidrug-resistant strains such as *Candida auris*, now recognized as a global health threat (Clancy and Nguyen, 2016; Lockhart et al., 2017; Rhodes and Fisher, 2019).

**Figure 2:**
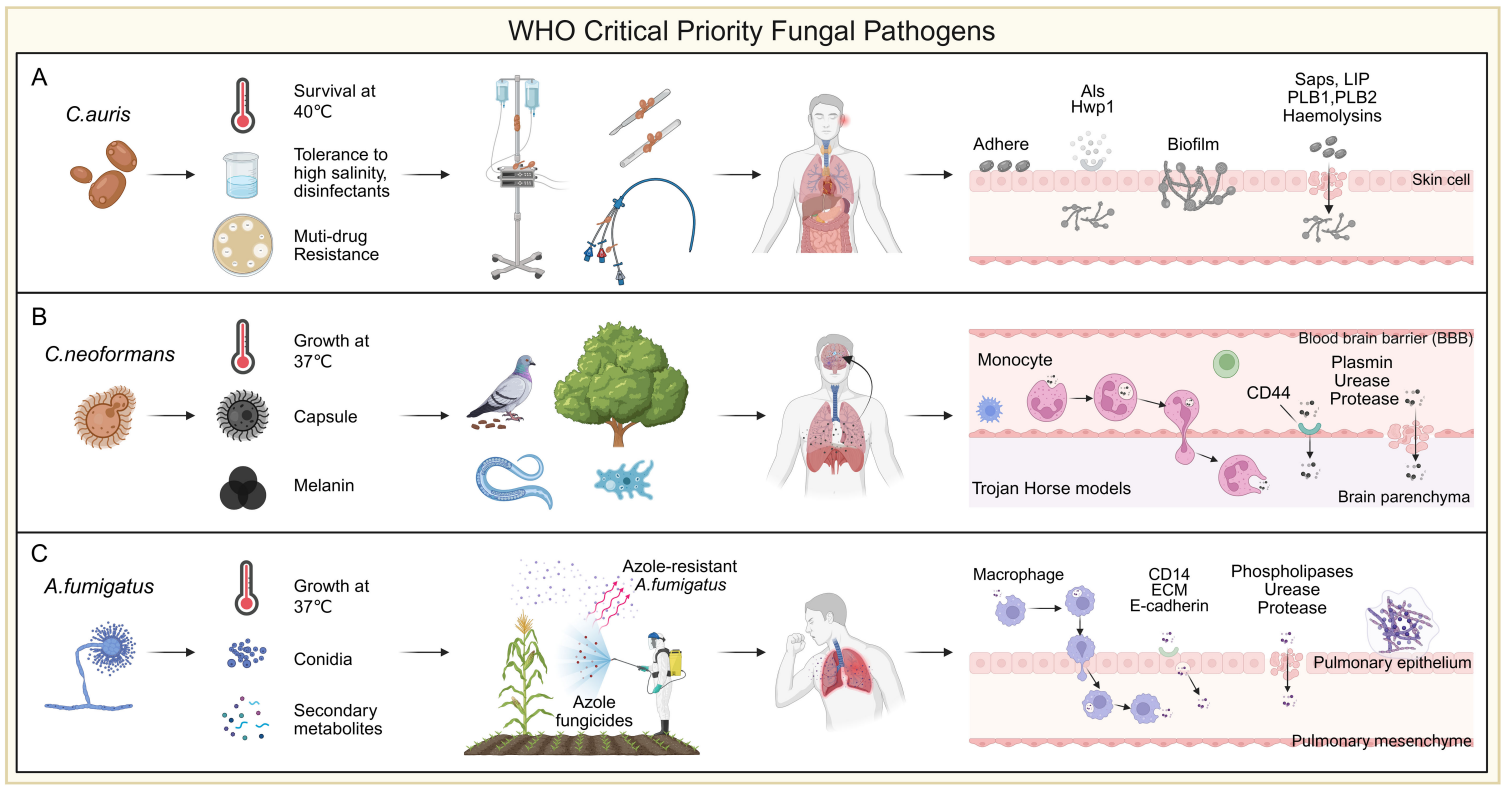
WHO critical priority fungal pathogens. **(A)** *Candida auris* is a multidrug-resistant fungus that can survive at 40 °C, tolerate high-salt conditions, and resist common disinfectants. A key risk factor for infection is its ability to adhere to abiotic surfaces, particularly medical devices such as ventilator tubes, central lines, feeding tubes, and urinary catheters. Its strong biofilm-forming capacity on these surfaces and skin promotes persistent colonization and increases the risk of invasive infection. Initial adhesion is mainly mediated by the Als family and Hwp1, while tissue invasion involves enzymes such as Saps, Plb1/2, Lip, and hemolysins. **(B)** *Cryptococcus* species produce classical virulence factors such as capsule and melanin, and exhibit thermotolerance at 37 °C. Interactions with environmental hosts like pigeons and amoebae have contributed to their resistance to heat and phagocytosis. *C*. *neoformans* displays notable neurotropism, disseminating from the lungs and crossing the blood-brain barrier (BBB) to cause meningoencephalitis. Translocation occurs via endothelial transcytosis or a “Trojan horse” mechanism mediated by monocytes. Inhaled spores are taken up by circulating monocytes, transported to the brain, and released into the parenchyma. Fungal cells also attach to the endothelium via CD44 to cross into tissue. To facilitate CNS invasion, *C*. *neoformans* secretes enzymes such as fibrinolysin, urease, and proteases that degrade host barriers. **(C)** *Aspergillus fumigatus* is a widespread environmental mold, with optimal growth at 37 °C. Triazole fungicides, structurally similar to medical azoles, can select for resistant strains in the environment. Inhalation of such strains by susceptible individuals may lead to azole-resistant infections. Humans inhale 100-1,000 conidia daily, typically cleared by the mucociliary system and alveolar macrophages. In immunocompromised hosts, conidia may evade clearance, germinate, and cause invasive disease. Surviving conidia can cross epithelial barriers via macrophage-mediated translocation or direct epithelial uptake involving CD14, ECM, and E-cadherin. Secreted phospholipases, ureases, and proteases further disrupt epithelial integrity, promoting tissue invasion.

Among *Candida* species, *C. albicans* remains the most prevalent, followed by *C. parapsilosis*, *C. glabrata and C. tropicalis*. These non-*albicans* species are showing rising resistance, particularly to azoles like fluconazole, complicating treatment and increasing the risk of clonal outbreaks (Lass-Flörl et al., 2024; Khan et al., 2025). A 10-year study at Duke University (2001–2010) reported an increase in *C. glabrata* resistance to echinocandins from 4.9% to 12.3%, and fluconazole resistance rates from 18% to 30% (Alexander et al., 2013). In Europe, from 2016 to 2022, echinocandin resistance in 15,400 *C. glabrata* isolates ranged from 1.5% to 12% (Rodríguez-Cerdeira et al., 2025). Globally, fluconazole resistance in *C. parapsilosis* had exceeded 10% before 2019. Since 2020, echinocandin and multidrug-resistant *C. parapsilosis* strains have been increasingly reported (Daneshnia et al., 2023). A multicenter study across 11 hospitals in China (over three years, 1,072 non-*albicans Candida* isolates) found that *C. tropicalis* exhibited a 7.1% resistance rate to both fluconazole and voriconazole, while *C. glabrata* showed 14.3% resistance to fluconazole and 11.6% cross-resistance to voriconazole (Xiao et al., 2015). *Candida auris* has drawn considerable attention due to its high level of multidrug resistance, with fluconazole resistance detected in 70%-90% of isolates (Navalkele et al., 2017; Ostrowsky et al., 2020). Its tolerance to 10% NaCl and quaternary ammonium disinfectants enhances its persistence in healthcare settings (Ahmad and Alfouzan, 2021) (**Supplementary File 1**). Most notably, unlike other *Candida* species, *C. auris* displays a remarkable ability to colonize abiotic surfaces (Santana et al., 2023). This trait facilitates its presence on a wide range of medical equipment, including catheters, ventilators, and surgical tools, contributing to nosocomial transmission (Haq et al., 2024). Skin colonization by *C. auris* is a known risk factor for bloodstream infections (BSIs), with approximately 5%-10% of colonized individuals developing fungemia (Lyman et al., 2023) (**Figure 2**).

In recent years, *Candida*-related breakthrough BSIs have emerged as complex clinical challenges, often occurring despite standard antifungal therapy. These infections are mainly attributed to *C. glabrata, C. krusei, C. parapsilosis*, and *C. tropicalis (* Zhai et al., 2020; Puerta-Alcalde et al., 2023). Recent reviews indicate that such breakthrough infections are strongly associated with antifungal resistance (Kontoyiannis et al., 2010; Fisher et al., 2019; Jenks et al., 2020). Notably, beyond classical resistance mechanisms, heterogeneous resistance has been implicated in *C. parapsilosis* breakthrough infections during echinocandin prophylaxis, suggesting more nuanced resistance dynamics (Zhai et al., 2024).

### 2.2 Cryptococcus

Cryptococcosis is a life-threatening invasive fungal infection primarily caused by *Cryptococcus neoformans* and *Cryptococcus gattii*, accounting for approximately 152,000 new cases and 110,000 deaths annually, predominantly among immunocompromised individuals such as HIV/AIDS patients and organ transplant recipients (Chayakulkeeree and Perfect, 2006; Kwon-Chung et al., 2014). *Cryptococcus* species are widely distributed in nature, and their environmental adaptation strategies have facilitated the evolution of traits enabling human infection. Natural hosts such as pigeons, amoebae, and nematodes have contributed to the development of thermotolerance and resistance to phagocytosis in *Cryptococcus (* May et al., 2016 *)*. Notably, *C. neoformans* exhibits pronounced neurotropism and frequently invades the central nervous system (Chen et al., 2022). Both *C. neoformans* and *C. gattii* disseminate from the lungs and cross the blood-brain barrier (BBB) to cause meningoencephalitis (Kronstad et al., 2011) (**Figure 2**). Fungal cells penetrate the BBB either through transcytosis across endothelial cells lining cerebral vessels or via a “Trojan horse” mechanism involving carriage within phagocytes, ultimately leading to life-threatening meningoencephalitis (Kronstad et al., 2011). Globally, nearly 250,000 cases occur each year, and without timely intervention, the mortality rate approaches 100% (Iyer et al., 2021). Current treatment options are limited to three major classes of antifungal agents. Azoles, especially fluconazole, are widely used for consolidation and maintenance therapy. However, resistance to fluconazole in emerging *Cryptococcus* strains is on the rise (Ngan et al., 2022). Echinocandins are largely ineffective against *Cryptococcus*, and the efficacy of other agents is often compromised by host toxicity or fungal resistance (Denning, 2003) (**Supplementary File 1**). Amphotericin B (AmB), the only approved fungicidal agent for cryptococcosis, targets ergosterol in the fungal membrane and remains a cornerstone of induction therapy. However, despite its effectiveness, AmB often fails to achieve complete fungal clearance, and relapses are common (Guidelines for The Diagnosis, Prevention and Management of Cryptococcal Disease in HIV-Infected Adults, Adolescents and Children: Supplement to the 2016 Consolidated Guidelines on the Use of Antiretroviral Drugs for Treating and Preventing HIV Infection, 2018). Its clinical utility is further limited by severe toxicity and restricted availability due to economic and logistical barriers (Perfect et al., 2010). Recent studies highlight the role of drug tolerance and persistence in cryptococcosis (Chen et al., 2024; Ke et al., 2024). Unlike genetic resistance, these phenotypes enable fungal cells to survive high concentrations of antifungals without a measurable increase in minimum inhibitory concentration (MIC), contributing to chronic and relapsing infections (Berman and Krysan, 2020). For example, during pulmonary infection, *Cryptococcus* can enter a quiescent state that confers high tolerance to AmB (Ke et al., 2024). Upon CNS invasion, activation of the glucose repression regulator Mig1 has been linked to enhanced AmB tolerance (Chen et al., 2024). These mechanisms significantly reduce AmB efficacy in animal models of cryptococcal meningitis. A growing hypothesis proposes that drug tolerance and persistence may precede and facilitate the development of stable resistance, as observed in bacterial pathogens. Despite their clinical implications, these non-classical mechanisms of antifungal failure remain underexplored and warrant further investigation to inform new therapeutic strategies.

### 2.3 Aspergillus

Invasive aspergillosis (IA) is one of the most common fungal infections in immunocompromised hosts, including invasive pulmonary aspergillosis, sinusitis, disseminated aspergillosis, and infections affecting individual organs (Schauwvlieghe et al., 2018). In the European Union alone, more than 2.25 million people suffer from infections caused by *Aspergillus (* Prevention, E. C. f. D. & Control, 2013). Unfortunately, recent studies have reported global emerging resistance to azole antifungals in clinical and environmental isolates (Barber et al., 2021; Rhodes et al., 2022) (**Supplementary File 1**). Azole resistance in *A. fumigatus* can arise via two main routes. In the clinical setting, prolonged azole exposure in patients undergoing antifungal therapy may lead to the selection of resistant strains that persist despite treatment and continue to cause infection (Howard et al., 2009). Alternatively, in the external environment, *A. fumigatus* strains residing on decaying plant material may be exposed to azole-based agricultural fungicides, which share structural and functional similarities with medical azoles (Prigitano et al., 2019; Schoustra et al., 2019; Pontes et al., 2020). This environmental route has been increasingly recognized as a major driver of resistance development (Snelders et al., 2009; Verweij et al., 2020). Notably, these resistance mechanisms have been shown to spread globally via horticultural products, particularly plant bulbs, and the airborne dissemination of conidia is uncontrollable (Dunne et al., 2017). Humans inhale an estimated 100-1,000 *Aspergillus* conidia daily, most of which are cleared by the mucociliary system of airway epithelial cells and resident alveolar macrophages (van de Veerdonk et al., 2017). However, in immunocompromised individuals, conidia that escape clearance can persist, germinate, and initiate invasive infection (Margalit and Kavanagh, 2015; van de Veerdonk et al., 2017) (**Figure 2**).

Effective treatment measures for IA include optimized prevention, timely diagnosis, and early antifungal therapy, which may also involve immunomodulation and surgery. The development of new antifungal drugs for aspergillosis includes Re-zafungin (CD101-IV), a novel echinocandin with unique pharmacokinetic properties that allows for weekly dosing and shows effective *in vitro* and *in vivo* activity against multiple *Aspergillus* species (Sandison et al., 2017; Wiederhold et al., 2018); Fosmanogepix (E1210/APX001), a broad-spectrum antifungal agent with a novel mechanism of action (inhibition of fungal glycosylphosphatidylinositol-insulin glucose biosynthesis), which has shown efficacy in IA animal models (Shaw and Ibrahim, 2020); Ibrexafungerp (SCY-078), a new class of unique glucan synthase inhibitors (triterpene compounds) (Rivero-Menendez et al., 2021); and Olorofim (F901318), a broad-spectrum antifungal agent with fungal-specific inhibition (Maertens et al., 2025). Voriconazole, isavuconazole, and posaconazole are substrates and inhibitors of CYP3A4 (Townsend et al., 2017). Long-term use of carbamazepine, phenytoin, and rifampin can significantly reduce the steady-state plasma concentrations of these drugs, leading to treatment failure. Additionally, voriconazole is also a substrate and inhibitor of CYP2C19, and glucocorticoids (CYP2C19 inducers) and CYP2C19 gene polymorphisms can influence its metabolism (Tian et al., 2021). Genomic epidemiology methods further suggest a potential link between the increasing clinical incidence of azole-resistant IA and the increasingly widespread presence of azole-resistant genotypes in environmental isolates (Cao et al., 2021).

## 3 Common antifungal drugs and associated resistance mechanisms

**Figure 3:**
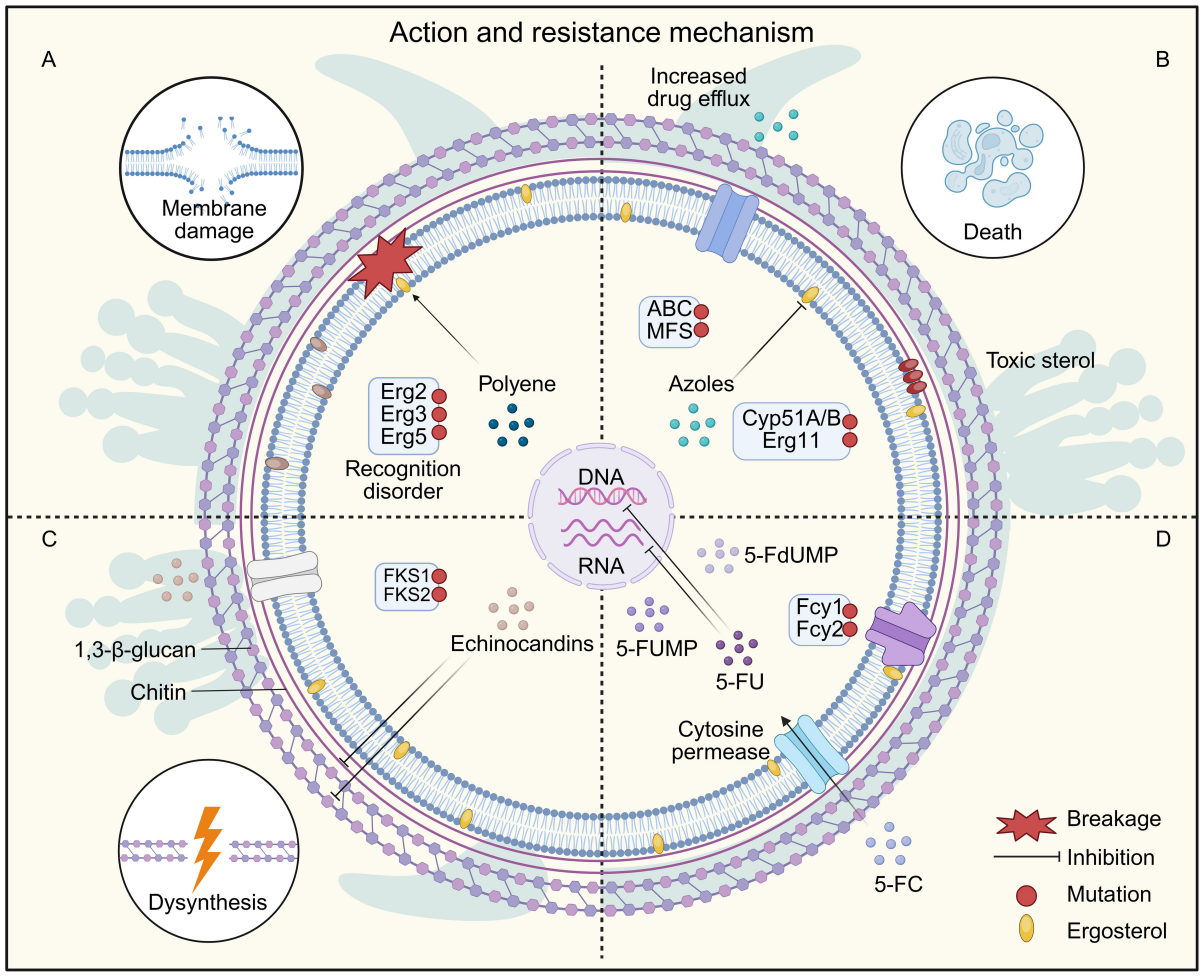
Fungal Resistance Mechanisms of Common Antifungal Drugs. **(A)** Polyene drugs cause cell death by inhibiting ergosterol synthesis and forming ion pores on the cell membrane. Resistance to polyenes is mainly caused by mutations in ergosterol biosynthesis genes, which deplete the target ergosterol, leading to the production of alternative sterols that do not interact with polyenes. **(B)** Azole drugs exert their antibacterial activity by inhibiting lanosterol 14-α-demethylase (encoded by *ERG11* in *Candida* and *CYP51A/B* in *Aspergillus*), and blocking the ergosterol synthesis and accumulation of toxic sterols. Resistance to azole drugs occurs mainly through mutations in the drug target, resulting in reduced drug-binding affinity, and also through overexpression of the drug target through the transcriptional activator Upc2. In addition, overexpression of ABC/MFS efflux pumps is also involved in drug resistance. **(C)** Echinocandin prevents 1,3-β-D-glucan and chitin biosynthesis by inhibiting 1,3-β-glucan synthase and chitin synthase, thereby causing loss of cell wall integrity and cell wall stress. Resistance to echinocandin mainly involves mutations in the genes encoding the drug target *FKS*. **(D)** 5-FC enters the cell by cytosine permease, interferes with RNA and DNA synthesis after conversion to 5-FU by cytosine deaminase. 5-FU is converted to 5-FdUMP, thereby inhibiting thymidylate synthesis and downstream DNA biosynthesis. 5-FU is also converted to 5-FUMP by UPRT, inhibiting RNA interference to translate proteins. Resistance to 5-FC mainly involves mutations in the cytosine permeases Fcy1 and Fcy2.

Common antifungal drugs include polyenes, azoles, allylamines, morpholines, and echinocandins, all of which function as antimetabolic agents targeting essential fungal structures or biosynthetic pathways (Fisher et al., 2022; Vitiello et al., 2023). Their mechanisms of action are summarized in **Figure 3**, and the classification, molecular targets, and known resistance mechanisms are listed in **Table 1**. Fungal responses to antifungal agents typically fall into three categories: resistance, tolerance, and persistence. Additionally, many intracellular or latent fungal cells can enter a dormant state, resulting in downregulation of drug targets, reduced membrane permeability, and decreased susceptibility to treatment (Arastehfar et al., 2023). These adaptations significantly limit the efficacy of antifungal therapy. For example, phagocytosis of fungal cells can be influenced by macrophage surface receptors interacting with fungal ligands, affecting drug access and immune clearance (Uribe-Querol and Rosales, 2020). Clinically, antifungal resistance has become an emerging challenge with both spatial and temporal dimensions (Fisher et al., 2018; Lockhart et al., 2023). Notable examples include resistant variants of *Aspergillus fumigatus* and *Candida auris*, a multidrug-resistant species that has spread globally (Chowdhary and Meis, 2018; Rhodes and Fisher, 2019). The vast diversity of fungal species and the evolutionary pressures driving resistance highlight the unpredictable nature of future fungal threats. Therefore, continuous surveillance and rapid response are critical to mitigating the growing burden of antifungal resistance.

**Table 1:** Research progress of new antifungal drugs.

Classification	Target	New drug	Experimental fungi	Latest study phase in	Ref.
Triazole	Ergosterol Fungal cytochrome P450 family member CYP51A1 protein Lanosterol 14α demethylase	Opelconazole (PC945)	*Cryptococcus*	III	(Cass et al., 2021; Ito et al., 2021)
			*Aspergillus*		
		Oteseconazole	*Candida albicans*	III completed	(Fothergill et al., 2014; Hoenigl et al., 2021; Sobel and Nyirjesy, 2021; Martens et al., 2022)
		Oteseconazole(VT-1161)	*Candida albicans*		(Martens et al., 2022; Wang et al., 2024)
		Quilseconazole(VT-1129)	*Candida auris*	III	(Treviño-Rangel et al., 2022)
		TFF voriconazole	*Aspergillus*	II	(Sigera and Denning, 2023)
Echinocandin	Cell wall Beta-1, 3-glucan synthase related proteins	Ibrexafungerp In VVC	*Candida albicans*	III completed	(Schwebke et al., 2022; Goje et al., 2023)
		Ibrexafungerp In IC/Candidemia	*Candida albicans*	III ongoing	(Spec et al., 2019; Sobel et al., 2022)
		Ibrexafungerp	*Candida auris*	III	(Arendrup et al., 2020)
			*Aspergillus*	IIb	(Petraitis et al., 2020; Angulo et al., 2022)
		Rezafungin(CD101)	*Candida albicans*	III completed	(Thompson et al., 2023)
			*Candida auris*	III	(Helleberg et al., 2020; Kovács et al., 2021; Thompson et al., 2021; Thompson et al., 2023)
			*Aspergillus*	III	(Helleberg et al., 2020)
Gwt-1 inhibitor	Phosphatidylinositol glycan anchor biosynthesis class W(Gwt-1) protein inhibitor	Fosmanogepix(Prodrug of APX001A N-phosphonyl oxymethyl)	*Candida albicans*	II completed	(Pappas et al., 2023)
			*Candida auris*	II	(Vazquez et al., 2023)
			*Cryptococcus*	None	(Petraitiene et al., 2021; Hodges et al., 2023)
			*Aspergillus*	II	(Gebremariam et al., 2022)
		Manogepix(APX001A)	*Candida* *Aspergillus* *Cryptococcus* Some rare molds	II	(Kapoor et al., 2019; Gintjee et al., 2020; Maphanga et al., 2022; Pfaller et al., 2022)
Chs competitive inhibitor	Cell wall chitin synthase protein	Nikkomycin Z	*Saccharomyces cerevisiae*	II (early termination)	(Wu et al., 2022)
Polyene (encochleated AMB)	Cell wall ergosterol	Oral lipid nanocrystal amphotericin B (MAT 2203)	*Cryptococcus*	III	(Boulware et al., 2023)
			*Aspergillus*	I	(Kriegl et al., 2025)
Antimicrobial peptide	Protein killer-resistant 9 (KRE9), a β-1,6-glucan synthase	CGA-N12	*Candida albicans*	Experimental phase	(Li et al., 2018; Li X. et al., 2022; Li R. et al., 2023)
Eterocyclic compounds thiazolylhydrazones	Fungal antioxidant system	RN104-SEDDS	*Candida albicans*	Experimental phase	(Silva et al., 2024)
Orotomide	Inhibit pyrimidine synthesis (dihydroorotic dehydrogenase)	Olorofim (F901318)	*Aspergillus*	III	(Wiederhold, 2020; Escribano et al., 2022; Egger et al., 2023; Su et al., 2023; Feuss et al., 2024)
Non-ribosomally synthesized cyclic hexapeptide	Transported by Sit1 transporter (The mechanism and target are unknown)	BAL2062(GR-2397)	*Aspergillus*	I	(Shaw, 2022)
Arylpyrimidine derivatives	Disrupting the fungal mitochondrial membrane protein	T-2307	*Candida* *Cryptococcus*	Experimental phase	(Mitsuyama et al., 2008; Nishikawa et al., 2010; Yamashita et al., 2019; Wiederhold et al., 2020; Wiederhold, 2021)

### 3.1 Mechanisms of resistance to cell wall-targeting antifungals: echinocandins

Antifungal agents targeting the fungal cell wall exert their effects by disrupting the biosynthesis of essential structural components. Among them, echinocandins, such as caspofungin, are the most clinically advanced class. These agents are cyclic hexapeptides with lipid side chains that inhibit 1,3-β-D-glucan synthase, an enzyme critical for glucan synthesis and fungal cell wall integrity (Chen et al., 2011). Echinocandins exhibit fungicidal activity against *Candida* species and fungistatic activity against *Aspergillus* spp. However, they have limited or no efficacy against certain emerging *Candida* species, such as *Candida auris* and *Candida parapsilosis*, highlighting a growing concern regarding their spectrum of activity (Roemer and Krysan, 2014). Other antifungal agents, including polyoxins and nikkomycins, act as chitin synthase inhibitors (Li et al., 2011). These compounds are structural analogs of chitin synthase substrates and competitively inhibit enzyme activity, thereby disrupting chitin biosynthesis and impairing fungal cell wall construction (Jackson et al., 2013). Although promising *in vitro*, their clinical application remains limited, and further development is required to evaluate their therapeutic potential. The primary mechanism of resistance to echinocandins involves point mutations in the *FKS1* and *FKS2* genes, which encode subunits of 1,3-β-D-glucan synthase (Deane, 2023; Hu et al., 2023; Li J. et al., 2025; Zajac et al., 2025). These mutations reduce drug binding affinity and are associated with treatment failure in invasive candidiasis (Schikora-Tamarit and Gabaldón, 2024; ElFeky et al., 2025; Zajac et al., 2025).

### 3.2 Mechanisms of resistance to membrane-targeting antifungals: polyenes and azoles

#### 3.2.1 Polyene antifungal antibiotics interact with cell membrane sterols

Polyene antifungal agents—such as nystatin, AMB, and natamycin—are broad-spectrum drugs commonly used to treat opportunistic fungal infections caused by *Candida*, *Cryptococcus*, *Aspergillus*, and *Lentinus* species (Carolus et al., 2020; Tugume et al., 2023; Lass-Flörl et al., 2024; Vishwakarma M and Soni, 2024). AMB exerts its antifungal activity through a dual mechanism. Their primary mode of action involves binding to ergosterol, a key sterol component of fungal cell membranes, leading to the formation of pores that disrupt membrane integrity and cause leakage of intracellular ions such as Na^+^, K^+^, H^+^, and Cl^-^, ultimately inhibiting fungal growth (Gray et al., 2012; Wang et al., 2021; Lee et al., 2023). In addition to membrane disruption, AMB induces an oxidative burst by promoting the generation of reactive oxygen species (ROS) within fungal cells. This ROS production, linked to mitochondrial respiratory chain dysfunction, leads to oxidative damage of critical cellular components including membranes, mitochondria, proteins, and DNA (Mesa-Arango et al., 2014; Singh et al., 2017). The combined effects of ionic imbalance and elevated ROS levels cause multiple deleterious impacts culminating in fungal cell death (Phillips et al., 2003; Sangalli-Leite et al., 2011; Mesa-Arango et al., 2014). Unlike protein-based targets, ergosterol is not gene-encoded, making polyene resistance relatively rare. However, when resistance does occur, such as in *Candida albicans* or *Aspergillus*, it is typically associated with mutations in the ergosterol biosynthesis pathway, particularly in genes such as *ERG2*, *ERG3*, *ERG5* and *ERG11 (* Bhattacharya et al., 2018). These mutations lead to altered membrane sterol composition, including depletion or substitution of ergosterol, reducing drug binding and effectiveness (Cannon et al., 2009; Jensen et al., 2015). Despite their potency, polyenes, especially AMB, are associated with significant toxicity, including nephrotoxicity, infusion-related reactions (e.g., fever, chills), and venous irritation at the injection site (Deray, 2002; Kagan et al., 2012; Lakhani et al., 2019; Wang et al., 2021). Moreover, their intravenous administration requirement limits outpatient and long-term use (Gray et al., 2012).

#### 3.2.2 Azole antifungal agents can inhibit cytochrome P450

Azoles are a major class of antifungal drugs characterized by a five-membered heterocyclic ring. Clinically relevant azoles include fluconazole, clotrimazole, miconazole, and ketoconazole, which are widely used for treating mucosal and cutaneous candidiasis as well as dermatophytosis, particularly in immunocompromised patients (Shapiro et al., 2011; Roemer and Krysan, 2014). Azoles exert their antifungal effect by inhibiting lanosterol 14-α-demethylase (Erg11), a cytochrome P450-dependent enzyme essential for ergosterol biosynthesis, thereby disrupting cell membrane integrity and function (Roemer and Krysan, 2014). However, azoles are less effective for aspergillosis or systemic yeast infections, and long-term use may result in hepatotoxicity, though they are generally better tolerated than polyenes (Benitez and Carver, 2019). Resistance to azoles is increasingly reported and involves several mechanisms: Overexpression of efflux pumps, including members of the ABC transporter superfamily and major facilitator superfamily (MFS); Point mutations or overexpression of *ERG11*, reducing azole binding to the target enzyme (Lee et al., 2021). These resistance mechanisms reduce intracellular drug accumulation and undermine treatment efficacy, posing a major challenge in the clinical management of fungal infections.

### 3.3 Mechanism of resistance to fungal nucleic acid synthesis: 5-fluorocytosine

Among antifungal antimetabolites, 5-fluorocytosine (5-FC) is the most prominent example. As a fluorinated analog of cytosine, it enters fungal cells and is converted intracellularly to 5-fluorouracil (5-FU), which inhibits DNA and RNA synthesis, thereby exerting antifungal effects (Lee et al., 2021). Despite its clinical utility, 5-FC is prone to rapid resistance development when used as monotherapy. The primary resistance mechanisms include mutations in *FCY2*, encoding cytosine permease (which mediates drug uptake), and mutations in *FCY1*, encoding cytosine deaminase (required for 5-FC activation) (Longley et al., 2003; Després et al., 2022). These mutations impair drug entry or metabolic conversion, leading to treatment failure. To enhance efficacy and reduce the risk of resistance, 5-FC is typically used in combination with AMB, especially in the treatment of cryptococcal meningitis (Bennett et al., 1979; Saag et al., 2000; Billmyre et al., 2020). This combination has demonstrated synergistic effects and remains a cornerstone of therapy for invasive cryptococcosis. Additionally, it shows activity against *Candida albicans* and certain saprophytic fungi (Hope et al., 2004; Papon et al., 2007).

## 4 A decade of progress: novel antifungal drugs targeting resistance

The main mechanism of antifungal action lies in inhibiting essential molecules, such as ergosterol (azole class) or 1,3-β-D-glucan (echinocandins), or by binding to ergosterol (polyene class), leading to the formation of pores and altering the integrity and permeability of the cell membrane, thereby affecting the membrane or fungal cell wall.

Despite the limited number of targets and the emergence of resistance, which pose challenges for antifungal therapy, new drugs such as Ibrexafungerp (formerly known as SCY-078), Rezafungin, Fosmanogepix and Olorofim have shown promising clinical efficacy (Arendrup et al., 2020; Wiederhold, 2020; Kovács et al., 2021; Thompson et al., 2021; Thompson et al., 2023; Vazquez et al., 2023; Feuss et al., 2024). Currently, marketed antifungal drugs have undergone extensive structural modifications and modifications. After thorough safety and efficacy evaluations, along with *in vivo* and *in vitro* model studies, the most promising antifungal compounds in preclinical and clinical development include novel triazoles, glucan synthase inhibitors, and small-molecule polypeptides. We have collected the latest 10 years’ developments in the development of novel antifungal drugs targeting four common pathogenic fungi: *Candida albicans*, *Candida auris*, *Cryptococcus*, and *Aspergillus* (**Table 1**).

### 4.1 Antifungal drugs targeting the cell wall

Recent advances in antifungal therapy have introduced new agents targeting the fungal cell wall, notably Ibrexafungerp and Rezafungin, both of which have shown promising results in clinical trials against *Candida albicans*, *Candida auris*, and *Aspergillus* spp (Spec et al., 2019; Arendrup et al., 2020; Petraitis et al., 2020; Angulo et al., 2022; Schwebke et al., 2022; Sobel et al., 2022; Goje et al., 2023; Thompson et al., 2023). These agents represent novel approaches in overcoming limitations of traditional antifungals, particularly in addressing resistance and improving patient compliance. Ibrexafungerp (formerly SCY-078/MK-3118, brand name Brexafemme) is a first-in-class oral β-(1,3)-D-glucan synthase inhibitor (GSI) and a fourth-generation triterpenoid antifungal. It offers a broad-spectrum activity against multiple *Candida* species, including strains resistant to azoles and echinocandins, as well as activity against *Aspergillus*, *Penicillium variotii*, and some rare dimorphic fungi (Spec et al., 2019; Arendrup et al., 2020; Petraitis et al., 2020; Angulo et al., 2022; Sobel et al., 2022). Preclinical studies also suggest potential efficacy against *Pneumocystis jirovecii (* El Ayoubi et al., 2024). Its dual route of administration, oral and intravenous, provides dosing flexibility, which is particularly beneficial for long-term outpatient management. Additionally, Ibrexafungerp maintains efficacy against *Candida* strains harboring *FKS* mutations, reducing the risk of cross-resistance with echinocandins (Nunnally et al., 2019). Its high protein-binding capacity and preferential accumulation in vaginal tissues make it especially suitable for treating vulvovaginal candidiasis (Schwebke et al., 2022; Goje et al., 2023; Kow et al., 2025). However, it has limited activity against *Mucorales* and *Fusarium* spp., and its relatively low oral bioavailability (~50%) may impact systemic efficacy (Lamoth and Alexander, 2015). Long-term safety data are also still being collected. Rezafungin (formerly CD101) is a next-generation echinocandin with structural modifications that confer a long half-life (~133 hours), enabling once-weekly intravenous administration. This feature greatly enhances treatment convenience and adherence, especially in outpatient or maintenance settings. FDA-approved for the treatment of candidemia and invasive candidiasis, Rezafungin demonstrates strong efficacy against *Candida* spp., including *C. auris*, with a well-documented safety profile (Thompson et al., 2024b). However, its spectrum of activity is narrower than that of Ibrexafungerp, with limited data on efficacy against molds or rare fungal pathogens (Fung and Shirley, 2025). Additionally, its intravenous-only formulation restricts its use in home-based care or resource-limited settings. There is currently a lack of clinical data for infections such as endocarditis, osteomyelitis, and meningitis caused by *Candida*. Another promising agent is Fosmanogepix, a first-in-class Gwt1 enzyme inhibitor that targets glycosylphosphatidylinositol (GPI) anchor biosynthesis, a process essential for fungal cell wall integrity, adhesion, and virulence (Wiederhold, 2020; Escribano et al., 2022; Egger et al., 2023; Vazquez et al., 2023; Feuss et al., 2024). By inhibiting Gwt1, Fosmanogepix disrupts the anchoring of mannoproteins on the fungal surface, impairing growth and pathogenicity through a mechanism distinct from azoles and echinocandins, thereby reducing the likelihood of cross-resistance (Pappas et al., 2023). It exhibits potent *in vitro* activity against a broad range of pathogens, including *Candida* spp. (except *C. krusei*), *C. auris*, *Aspergillus* spp., and even *Mucorales*, a group notoriously resistant to conventional antifungals (Petraitiene et al., 2021; Hodges et al., 2023; Vazquez et al., 2023). Fosmanogepix also shows favorable pharmacokinetic properties, with >90% oral bioavailability unaffected by food intake and a half-life of approximately 2.5 days. Its ability to penetrate sanctuary sites such as the central nervous system and eyes further enhances its therapeutic potential (Hodges et al., 2024). Currently in Phase III trials, Fosmanogepix has demonstrated efficacy in early studies for invasive candidiasis and is considered a promising candidate for drug-resistant or difficult-to-treat infections. Together, these emerging agents represent a significant shift in antifungal therapy, expanding treatment options beyond traditional mechanisms, addressing current resistance gaps, and offering improved pharmacological and patient-centered profiles. Future research should continue to evaluate these novel agents in diverse clinical settings and against emerging fungal threats.

### 4.2 Antifungal drugs targeting the cell membrane

Among antifungal agents targeting the fungal cell membrane, Oteseconazole (VT-1161) has emerged as a promising novel triazole, with recent clinical and regulatory progress. It has successfully completed Phase III clinical trials and has been approved by the U.S. FDA for the treatment of recurrent vulvovaginal candidiasis (RVVC) (Fothergill et al., 2014; Hoenigl et al., 2021; Sobel and Nyirjesy, 2021; Martens et al., 2022; Wiederhold, 2022). Mechanistically, Oteseconazole, like other triazoles, inhibits 14α-demethylase in the fungal cytochrome P450 system, thereby disrupting ergosterol biosynthesis, an essential component of fungal cell membranes (Hoy, 2022). This inhibition compromises membrane integrity, alters permeability, and leads to leakage of intracellular contents, ultimately impairing fungal growth and replication. Compared to existing azoles, Oteseconazole demonstrates enhanced antifungal activity, including efficacy against azole-resistant strains of *Candida* and *Aspergillus* spp. It exhibits favorable pharmacokinetics, including a suitable half-life, wide tissue distribution, and effective tissue penetration, critical for eradicating infection at various anatomical sites. In preclinical and clinical studies, it has shown superior *in vitro* activity and consistent therapeutic effects in animal models. Clinically, Oteseconazole has shown significant benefits in RVVC, achieving symptom relief, fungal burden reduction, and lower recurrence rates compared to standard therapies (Sobel and Nyirjesy, 2021; Martens et al., 2022). Its less frequent dosing also improves patient compliance, making it a potentially superior alternative to traditional azoles in this indication. In terms of safety, clinical trials indicate good tolerability, with mild and transient adverse effects and a low incidence of serious reactions. However, like other azoles, Oteseconazole inhibits human cytochrome P450 enzymes, posing a potential risk of drug, drug interactions, which remains a limitation, especially in patients receiving multiple medications.

Although azoles remain central to antifungal therapy due to their broad spectrum and oral availability, the risk of resistance, drug interactions, and incomplete eradication calls for continued refinement. Oteseconazole provides a valuable step forward, but long-term studies are still needed to fully assess its safety, effectiveness across populations, and utility in additional indications beyond RVVC.

### 4.3 Antifungal drugs targeting organelles

Mitochondria are essential organelles in fungi, playing central roles in energy metabolism, the respiratory chain, redox homeostasis, and various biosynthetic pathways. Disruption of mitochondrial function has emerged as a promising antifungal strategy, with several novel compounds demonstrating efficacy through mitochondrial targeting. One such agent is Olorofim, a dihydroorotate dehydrogenase inhibitor currently in advanced clinical development, which impairs mitochondrial pyrimidine biosynthesis, leading to defective DNA synthesis and subsequent fungal cell death (Oliver et al., 2016). Another example is CGA-N12, a synthetic antimicrobial peptide that exerts potent antifungal effects against *Candida albicans* by inducing reactive oxygen species (ROS) accumulation and collapsing the mitochondrial membrane potential, ultimately triggering apoptosis (Li et al., 2018). ATI-2307 (T-2307), an aromatic amidine compound, represents a novel class of mitochondrial respiratory chain inhibitors (Yamashita et al., 2019). It selectively targets fungal mitochondrial complexes, blocking electron transport and disrupting the proton gradient across the inner membrane. This results in membrane potential dissipation, ATP synthase inhibition, and energy depletion, culminating in fungal growth arrest and cell death (Nishikawa et al., 2010). ATI-2307 has demonstrated strong *in vitro* activity against various *Candida* species, with preliminary evidence suggesting potential efficacy against other pathogenic fungi, including *Rhizopus arrhizus*, *Mucor racemosus*, *Scedosporium* spp., and *Trichosporon asahii*, although further studies are warranted. In addition to synthetic compounds, several natural products have shown mitochondrial-targeting antifungal activity. For instance, berberine selectively accumulates in fungal mitochondria, disrupts membrane potential, and binds subunits of complex I; it also interacts with the Mdr1p efflux pump, potentially reversing azole resistance in *C. albicans (* Tong et al., 2021). Other plant-derived compounds, such as citronellal and perillaldehyde, induce ROS overproduction, resulting in mitochondrial and DNA damage (Tian et al., 2016; Chen et al., 2020; Trindade et al., 2022; Venancio et al., 2025). Moreover, cyclooxygenase inhibitors, which suppress prostaglandin E2 synthesis, have been shown to reduce fungal biofilm formation, highlighting their potential as adjunctive agents in antifungal therapy (Abdelmegeed and Shaaban, 2013).

### 4.4 Antifungal drugs targeting metabolic pathways and enzymes

Fungal metabolism involves a wide array of biochemical pathways essential for growth, survival, and virulence, including N-acetylglucosamine utilization, trehalose metabolism, lipid biosynthesis, energy production, and intracellular transport (Pan et al., 2018; Wijnants et al., 2022). These metabolic processes present attractive targets for antifungal drug development due to their indispensable roles in fungal physiology and pathogenesis (Ramakrishnan et al., 2016). A growing number of intracellular enzymes have been identified as potential antifungal targets. For instance, AMP-17, a novel antifungal peptide, interferes with several critical metabolic pathways in *Candida albicans*, including oxidative phosphorylation, RNA degradation, and fatty acid metabolism, effectively suppressing fungal growth (Yang et al., 2022). Similarly, ApoB-derived peptides exhibit antifungal properties primarily by compromising cell membrane integrity in *C. albicans (* Dell'Olmo et al., 2021). Another promising compound, α-erythromycin myrrh (α-red myrrh), inhibits Δ;24-sterol methyltransferase, a key enzyme in ergosterol biosynthesis encoded by *ERG6*. This inhibition reduces ergosterol content in a dose-dependent manner, disrupts membrane integrity, and inhibits fungal proliferation (Jahanshiri et al., 2017). Notably, α-red myrrh may also exert indirect effects on fungal gene expression by modulating host signaling pathways such as NF-κB, p38, and JNK, which influence *ERG6* regulation, suggesting a multifaceted mechanism of action (Jahanshiri et al., 2017). In addition to these, several novel enzyme inhibitors have shown promise. (S)-2-amino-4-oxo-5-hydroxyvaleric acid (RI-331), a homoserine dehydrogenase inhibitor, acts through an enzyme-assisted suicide mechanism by irreversibly binding to and inactivating Hom6p, an enzyme involved in amino acid biosynthesis, ultimately leading to fungal cell death (Yamaki et al., 1992). Likewise, RI-331 exhibits selective antifungal activity against *C. albicans*, *C. tropicalis*, and *C. glabrata*, but not against *Aspergillus* species (Jacques et al., 2003; Kuplińska and Rząd, 2021). Importantly, some fungal-specific enzymes offer high target selectivity with minimal risk to the host. For example, class II fructose-1,6-bisphosphate aldolase (FBA-II) is found exclusively in fungi and is absent in animals and higher plants, making it an ideal candidate for developing targeted antifungal agents with reduced off-target toxicity (Han et al., 2017; Wen et al., 2022).

### 4.5 Antifungal drugs targeting iron transporters

Iron is essential for fungal growth and pathogenicity, but the host limits its availability to prevent infection (Choi and Bessman, 2025). This has led to the concept of iron hijacking as a novel antifungal strategy. One promising approach is disrupting siderophore (ferrifer) biosynthesis, which fungi rely on to acquire iron (Balhara et al., 2014; Choi and Bessman, 2025). Inhibitors targeting enzymes such as adenosine phosphate transferase, non-ribosomal peptide synthase (NRPS), polyketide synthase, and NRPS-independent siderophore synthases impair microbial iron uptake and enhance host-mediated clearance (Petrik et al., 2012; Leal et al., 2013; Süssmuth and Mainz, 2017; Qiao et al., 2023; Zhang L. et al., 2023).

Natural products are also being explored. Celastrol, derived from *Tripterygium wilfordii*, inhibits the flavin-dependent monooxygenase FerA, essential for siderophore synthesis in *Aspergillus fumigatus (* Sun et al., 2019). This inhibition disrupts L-ornithine hydroxylation, a critical step in siderophore production, resulting in iron starvation and suppressed fungal growth (Martín Del Campo et al., 2016; FA et al., 2025). This highlights celastrol as both a potential therapeutic and a new antifungal target. Another strategy involves siderophore-drug conjugates, which improve delivery of antifungal agents by hijacking the fungal iron uptake system. Conjugates like ferrimycin combine a siderophore with antifungal drugs (e.g., triazoles, echinocandins, or polyenes) (Lakshminarayanan et al., 2024). These agents specifically bind fungal iron transporters, enhancing drug targeting and reducing toxicity to host cells, thus improving both efficacy and safety. A leading compound in this class is GR-2397 (also known as ASP2397 or VL-2397), a cyclic hexapeptide developed by Gravitas Therapeutics (Nakamura et al., 2017; Kovanda et al., 2019; Shaw, 2022). It enters fungal cells via the SIT1 transporter, which is absent in humans, ensuring fungal specificity (Dietl et al., 2019; Nakamura et al., 2019). GR-2397 has shown rapid fungicidal activity in murine aspergillosis models, and Phase 1 clinical trials demonstrated it is safe and well-tolerated (Rubino et al.,; Arendrup et al., 2016; Mammen Mammen et al., 2019). Recognized by the FDA as a Qualified Infectious Disease Product (QIDP), orphan drug, and Fast Track agent, GR-2397 is set to enter Phase 2 trials in 2025. Finally, competitive iron chelators like lactoferrin and gallium reduce fungal biofilm formation by replacing iron (Fernandes and Carter, 2017; Bastos et al., 2019; Fernandes et al., 2020; Li F. et al., 2022). Biofilm thinning enhances the treatment of mucosal infections and complements conventional antifungal therapy. Overall, targeting iron acquisition mechanisms represents a powerful, fungus-specific therapeutic direction with multiple avenues for innovation.

### 4.6 Antifungal drugs targeting antioxidant defense systems

During infection, fungal pathogens are continuously exposed to oxidative stress generated by the host immune response (Yaakoub et al., 2022). To survive and establish infection, they have developed a robust antioxidant defense system, including catalases, superoxide dismutases (SODs), glutathione peroxidases (GPxs), and peroxiredoxins (Leal et al., 2012; Arastehfar et al., 2023). These enzymes work synergistically to eliminate reactive oxygen species (ROS) and maintain redox homeostasis. Recent studies have highlighted their critical roles in fungal virulence and identified them as promising targets for antifungal therapy (Lionakis et al., 2023).

Amphotericin B (AMB), a widely used antifungal, can broadly induce reactive oxygen species (ROS) accumulation across 44 fungal species, including *Candida albicans*, *C. parapsilosis*, *C. glabrata*, *C. tropicalis*, *C. krusei*, *C.neoformans*, and *C. gattii (* Mesa-Arango et al., 2014). This oxidative stress is accompanied by the upregulation of genes encoding antioxidant proteins (Mesa-Arango et al., 2014). Correspondingly, AMB-resistant isolates often exhibit elevated catalase levels (Mesa-Arango et al., 2014). In *C. glabrata*, however, fluconazole-resistant strains harboring the Pdr1 P927L mutation show reduced catalase expression (Vermitsky and Edlind, 2004; Edlind and Katiyar, 2022). Similarly, in *Candida auris*, fluconazole resistance is associated with an adaptive trade-off: fluconazole-susceptible isolates display enhanced resistance to oxidative stress, whereas the majority (94.5%) of fluconazole-resistant strains exhibit reduced oxidative tolerance (Das et al., 2024). suggesting that catalase functions differently depending on the antifungal class involved.

Several compounds that target fungal antioxidant defenses have shown synergistic effects with existing antifungals. Cyclams, macrocyclic polyamines with antimicrobial activity, have demonstrated antifungal potential. For example, the cyclam salt H_4_[H_2_(^4-CF3^PhCH_2_)_2_Cyclam]Cl_4_ inhibits *C. albicans* biofilm formation and catalase activity, suppresses morphological transition, and reduces melanin production in *C. neoformans (* Cerqueira et al., 2024). SODs are also emerging attractive targets. Inhibitors such as N,N′-diethyldithiocarbamate (DDC) and ammonium tetrathiomolybdate (ATM) impair *C. albicans* biofilm formation and sensitize it to AMB (Seneviratne et al., 2008; De Brucker et al., 2013). Natural dihydroxybenzaldehydes (DHBAs), including 2,3- and 2,5-DHBA, inhibit SOD and glutathione reductase in *Candida* and *Cryptococcus* species, enhancing AMB efficacy (Kim et al., 2012b). Benzaldehyde and its analogs (e.g., *trans*-cinnamaldehyde, o-vanillin) inhibit filamentous fungi like *Aspergillus fumigatus* and act as chemosensitizers against *C. albicans* and *Candida auris* when combined with AMB, fluconazole, or itraconazole (Faria et al., 2011; Kim et al., 2011b).Phenolic compounds with redox-modulating activity also enhance antifungal action. Thymol (THY) disrupts fungal redox and ion homeostasis and synergizes with itraconazole against *A. fumigatus (* Kim et al., 2012a). Co-administration of THY with AMB sensitizes yeast pathogens including *C. albicans, C. tropicalis*, and *C. neoformans (* Kim et al., 2012a). Similarly, salicylaldehyde shows comparable effects (Kim et al., 2011a). Antioxidant defenses are equally critical in plant-pathogenic fungi (Park and Son, 2024). Phytic acid inhibits *Fusarium oxysporum* by compromising membrane integrity and suppressing antioxidant enzyme activity such as superoxide dismutase (SOD) and catalase (Li N. et al., 2023). Dehydroabietic acid (DHA), derived from rosin, inhibits the growth of multiple plant pathogens (such as *Alternaria alternata, Botrytis cinerea, Valsa mali, Pestalotiopsis neglecta, and F. oxysporum*) and reduces the activity of SOD, catalase, and peroxidase in *Alternaria alternata* (Chen et al., 2025).

Combining antioxidant-targeting agents with cell wall synthesis inhibitors has shown synergistic efficacy in model organisms, supporting combination therapies. However, due to the redundancy within antioxidant systems, complete inhibition via single targets remains challenging, and drug specificity must be optimized to avoid host toxicity. Targeting fungal redox homeostasis thus represents a promising strategy to overcome antifungal resistance.

### 4.7 Antifungal vaccines

Fungal vaccines offer a proactive strategy to prevent or control infections by stimulating the host immune system (Levitz and Golenbock, 2012). As antifungal resistance increases and limits the efficacy of conventional drugs, vaccines are emerging as promising adjuncts or alternatives (Dan and Levitz, 2006; Nami et al., 2019). Unlike single-target antifungals, vaccines trigger both T cell–mediated and antibody-based responses, enabling multi-pathway pathogen clearance (Cutler et al., 2007; Lionakis et al., 2023). These immune mechanisms are less susceptible to resistance mechanisms such as mutations, efflux pumps, and biofilm formation, making vaccines especially valuable against drug-resistant strains (Cassone, 2013; Sahu et al., 2022).

Several fungal vaccines have shown efficacy in preclinical studies, with some advancing to early clinical trials. Two recombinant *Candida* vaccines, PEV7 and NDV-3A, demonstrated safety and immunogenicity in Phase I trials. PEV7, which incorporates a truncated *C. albicans* Sap2 protein into influenza virosomes, protected rats from infection and induced memory B cell responses in human volunteers (De Bernardis et al., 2012; De Bernardis et al., 2018). A Sap2 vaccine derived from *C. parapsilosis* also conferred cross-species protection in *C. tropicalis*-infected mice (Shukla and Rohatgi, 2020). NDV-3A, based on the N-terminal region of *C. albicans* Als3 adhesin, elicited broad immunity and showed efficacy against recurrent vulvovaginal and oropharyngeal candidiasis (Spellberg et al., 2005; Spellberg et al., 2006). A Phase II study reported reduced recurrence of vulvovaginal candidiasis in women under 40 over 12 months (Edwards et al., 2018). NDV-3A also prevented kidney dissemination and catheter colonization in murine models, inhibited *C. auris* biofilm formation, enhanced macrophage phagocytosis, and improved micafungin efficacy (Alqarihi et al., 2019; Singh et al., 2019).

Targeting fungal cell wall polysaccharides is another promising strategy. These components activate complement and are recognized by receptors like Dectin-1, driving robust immune responses (Levitz, 2010). Vaccines against *Cryptococcus neoformans* capsule have been explored for over 40 years. Capsule-specific antibodies against the *Cryptococcus neoformans* improves survival, reduces fungal burden, and promotes granuloma formation in infected mice, limiting disease progression (Graybill et al., 1981; Dromer et al., 1987; Mukherjee et al., 1993b; Mukherjee et al., 1993a). These antibodies also enhance the efficacy of antifungals such as amphotericin B, fluconazole, and flucytosine, demonstrating synergistic effects in animal models and *in vitro* assays (Gordon and Lapa, 1964; Dromer and Charreire, 1991; Mukherjee et al., 1995; Feldmesser et al., 1996; Monari et al., 1999). The monoclonal antibody 18B7 completed Phase I trials in cryptococcal meningitis patients (Larsen et al., 2005). β-glucan conjugate vaccines (Levitz et al., 2015), such as Lam-CRM, a β-glucan–diphtheria toxin conjugate, and branched oligo-β-glucans, protect against systemic *Candida* and *Aspergillus* infections by enhancing phagocytosis and prolonging survival (Torosantucci et al., 2005; Bromuro et al., 2010; Liao et al., 2016). β-glucan particles (GPs), derived from *Saccharomyces cerevisiae*, also serve as effective antigen delivery systems and adjuvants (Huang et al., 2010; De Smet et al., 2013). Whole glucan particles (WGPs) conjugated with BSA have demonstrated protection against systemic aspergillosis and coccidioidomycosis (Clemons et al., 2014a; Clemons et al., 2015).

Pan-fungal vaccines can provide cross-protection against multiple fungal pathogens. Heat-killed *S. cerevisiae* (HKY) protects mice from infections caused by *Aspergillus, Coccidioides, Candida, Cryptococcus, Rhizopus*, and *Pneumocystis (* Stevens et al., 2011; Liu et al., 2012; Clemons et al., 2014b; Luo et al., 2014; Majumder et al., 2014; Martinez et al., 2017), and induces strong Th1 immune responses and antibodies against β-glucans and mannans (Liu et al., 2011). The peptide vaccine NXT-2 targets pathogens like *Candida*, *Aspergillus*, and *Pneumocystis*, shows protection in mice and primates (Rayens et al., 2021; Rayens et al., 2022; Wychrij et al., 2025). Deletion of the F-box protein Fbp1 in *C. neoformans*, part of the SCF(Fbp1) E3 ligase, triggers strong Th1-mediated immunity (Masso-Silva et al., 2018). Remarkably, heat-killed *fbp1Δ* cells confer cross-protection against diverse fungal pathogens, including *C. neoformans*, *C. gattii*, *Aspergillus fumigatus*, and *Candida albicans*, even in CD4^+^ T cell-deficient hosts, supporting their potential as a broad-spectrum vaccine (Wang et al., 2019).

mRNA-lipid nanoparticle (LNP) vaccines represent a novel platform. Fungal-targeted nanoconstructs (FTNx), using antisense oligonucleotides against *FKS1* and *CHS3*, can inhibit fungal cell wall synthesis genes, reduce fungal burden and improve survival in murine candidiasis models, exhibit broad-spectrum antifungal activity, including against clinical isolates of *Candida auris (* Chung et al., 2025). Additionally, CDA1-LNP, an mRNA-LNP vaccine effective against cryptococcosis in mice, has been shown to protect the majority of vaccinated animals from lethal infection (Li et al., 2025a).

Despite promising advances, challenges remain. Fungal similarity to human cells, immune evasion, and antigen variability complicate vaccine development. Practical hurdles such as storage, delivery, and competition with antifungals further limit progress (Oliveira et al., 2021; Loh and Lam, 2023). Future efforts will benefit from interdisciplinary approaches, novel platforms, and deeper insight into host–fungus interactions.

## 5 New strategies for drug development

**Figure 4:**
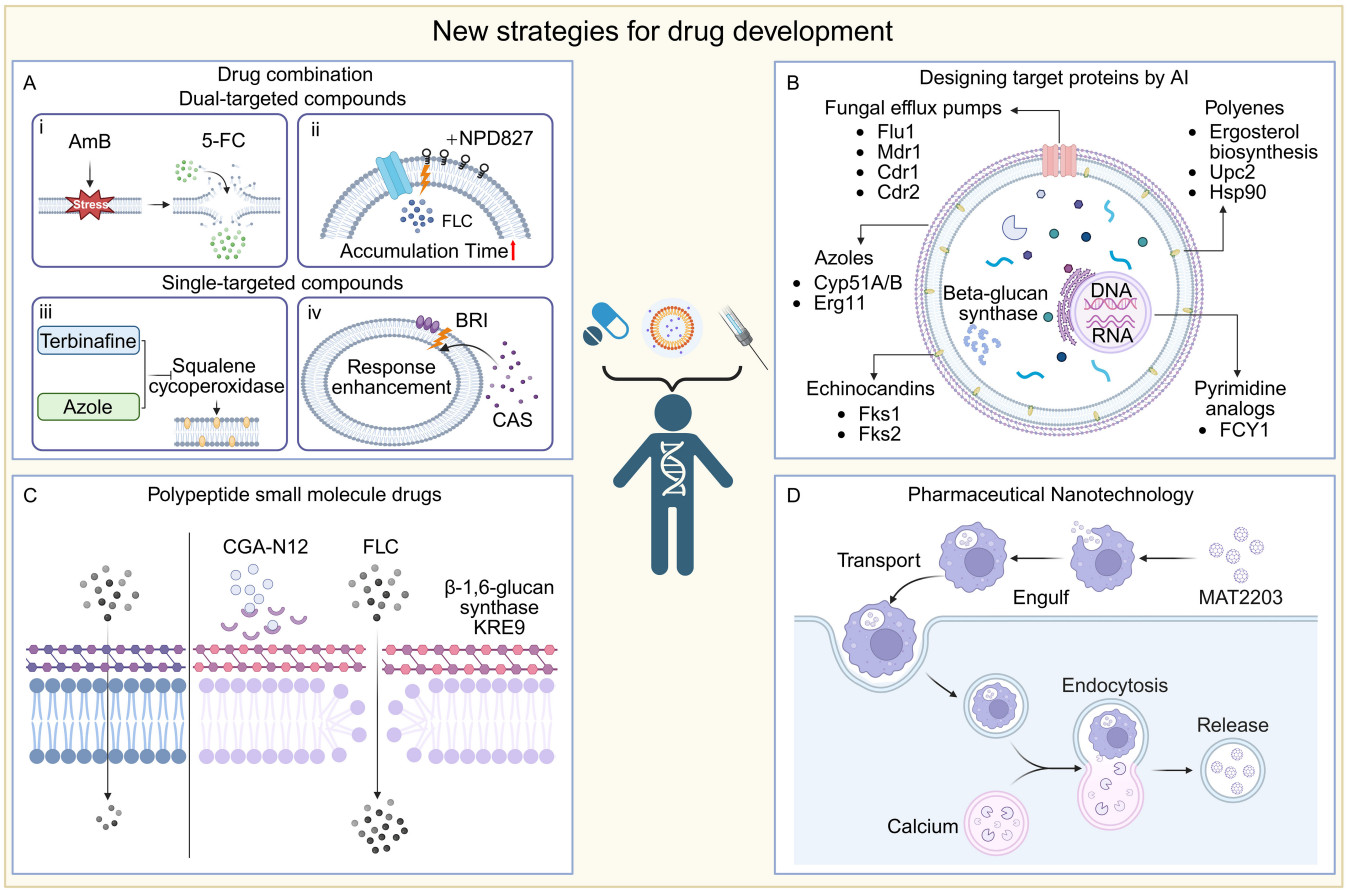
New strategies for drug development. **(A)** Combination: i) AMB disrupts fungal cell membrane integrity, thereby increasing intracellular 5-FC concentration and enhancing drug bioavailability; ii) Insertion of NPD827 into the cell membrane disrupts the efflux pump action and increases the accumulation time of the drug; iii) Squalene cycloperoxidase could be inhibited by terbinafine and azols, resulting in a dual inhibition of ergosterol biosynthesis; iv) The small molecule compound BRI enhances drug responsiveness to fungi by affecting the cell wall. **(B)** AI design: This figure shows the important target protein sites for the binding of drug small molecule compounds. **(C)** CGA-N12 inhibited the KRE9 target in β-1, 6-glucan synthase, disrupting the structural integrity of the fungal cell wall and improving the drug availability. **(D)** Pharmaceutical nanotechnology: an oral formulation of lipid nanocrystals, MAT2203, in which targeted cells (e.g., macrophages) swallow these nanocrystals and deliver them to the site of infection, where lower intracellular calcium concentrations trigger a nanocrystal-release mechanism that allows the drug to be released directly into the cell interior.

The emergence of resistance from the prolonged or monotherapeutic use of traditional antifungal agents, combined with the low success rate of new drug development, continues to hinder effective treatment of clinical fungal infections. Although repurposing existing drugs offers a cost-effective and time-efficient alternative for identifying new therapies, their effective concentrations—measured as the half-maximal inhibitory concentration (IC50)—often exceed the maximum safe plasma levels in humans, creating a major barrier to clinical application. Promisingly, combination therapy using drugs with different mechanisms of action can significantly lower the required dose of each agent, thereby reducing toxicity and limiting the likelihood of resistance development during treatment. At the same time, the development of novel antifungal agents remains a major research focus. Among recent advances, some efforts have centered on creating new formulations of existing effective drugs, including nanoparticle-based delivery systems, which improve drug solubility, bioavailability, and tissue targeting. Furthermore, novel small-molecule compounds such as cinnamaldehyde have shown antifungal activity through mechanisms like membrane disruption (Shreaz et al., 2016). In parallel, antimicrobial peptides targeting fungal-specific enzymes, such as chitin synthase, along with other innovative small molecules, are also under active investigation (**Figure 4**) (Ran et al., 2024).

### 5.1 Drug combination therapy

Combination therapy has gained importance in managing infectious diseases, especially amid rising antifungal resistance (Johnson and Perfect, 2007; Spitzer et al., 2017; Lee et al., 2021). It offers key benefits such as reducing resistance development, improving efficacy at lower doses, shortening treatment duration, and lessening toxicities like amphotericin B-associated nephrotoxicity (Bicanic et al., 2015; Tyers and Wright, 2019). By targeting multiple fungal pathways simultaneously, combinations can yield synergistic or additive effects, enhancing clinical outcomes.

The standard of care for cryptococcal meningitis demonstrates this approach, with AmB plus flucytosine (5-FC) or fluconazole as preferred regimens. A 7-day course of AmB (1 mg/kg/day) with 5-FC (100 mg/kg/day) yields the lowest 10-week mortality (24.2%), with 5-FC outperforming fluconazole (Day et al., 2013; Molloy et al., 2018). Liposomal AmB reduces toxicity and prolongs CNS exposure (Stone et al., 2016). A recent trial found that a single high-dose liposomal AmB with 5-FC and fluconazole reduced mortality and halved adverse events compared to WHO recommendations (Jarvis et al., 2022). In invasive aspergillosis (IA), azole–echinocandin combinations improve fungicidal activity and survival (Paulussen et al., 2014; Marr et al., 2015). with meta-analyses confirming benefits in salvage therapy (Panackal et al., 2014). Rising azole-resistant *Aspergillus fumigatus* underscores the need for novel agents, including ibrexafungerp, fosmanogepix, and olorofim, now in clinical trials.

Beyond conventional antifungal combinations, a host defense peptide mimetic has emerged as promising enhancers of existing antifungal agents. Brilacidin (BRI), a synthetic small molecule that recapitulates the amphipathic architecture of HDPs, augments caspofungin (CAS) activity against *Aspergillus fumigatus*, *Candida albicans*, *Candida auris*, and CAS-resistant *Cryptococcus* isolates (Dos Reis et al., 2023; Diehl et al., 2024; Dos Reis et al., 2024). Additionally, BRI potentiates azole efficacy by disrupting fungal cell wall integrity pathways and perturbing membrane potential (Dos Reis et al., 2023). These observations underscore the potential of BRI as an adjunctive therapy for recalcitrant fungal infections.

Another promising strategy involves combining antifungal agents with non-traditional pharmacological compounds. Heat shock protein 90 (Hsp90) acts as a central regulator of fungal stress responses, modulating resistance, morphogenesis, and virulence factor expression (Cowen et al., 2009). Inhibiting Hsp90 markedly reduces resistance to azoles and echinocandins, restoring susceptibility (Cowen and Lindquist, 2005; Singh et al., 2009; Lamoth et al., 2016). Radicicol and geldanamycin, both Hsp90 inhibitors, enhance azole efficacy by disrupting membrane integrity and capsule formation, impairing Hsp90 mitochondrial localization, and increasing reactive oxygen species in fungal pathogens (Cordeiro et al., 2016; Xiong et al., 2024). The echinocandin micafungin is also active against *Candida parapsilosis* isolates from neonates (da Silva et al., 2024). High-throughput screening identified clofazimine as a broad-spectrum synergist with fluconazole, caspofungin, and AmB, and others, enhancing activity against *Candida albicans*, *Cryptococcus neoformans*, and additional fungal pathogens (Robbins et al., 2015).

Immunomodulator-based combination strategies aim to both enhance host antifungal defenses and directly kill the pathogen, representing a frontier in antifungal therapy (Pirofski and Casadevall, 2006). Immunomodulatory combinations aim to boost host defenses while targeting the pathogen. Examples include the lectin pCramoll plus fluconazole, which improved survival and reduced fungal burden in *Cryptococcus gattii*–infected mice (Jandú et al., 2017); interferon-γ with AmB, which decreased cryptococcal CNS infections; and macrophage colony-stimulating factor with fluconazole, which enhanced macrophage fungicidal activity (Brummer and Stevens, 1994).

In summary, the growing array of antifungal combination therapies plays a vital role in overcoming resistance, enhancing efficacy, and expanding treatment options against invasive fungal infections. These approaches, from conventional drug combinations to novel immunomodulatory and non-antifungal partnerships, represent a promising advance in antifungal therapeutics.

### 5.2 Drug repurposing strategies

Drug repurposing, which involves applying approved or known safe drugs to entirely new therapeutic areas, aims to significantly shorten the research and development cycle, reduce costs, and quickly address clinical challenges caused by drug-resistant fungi (Perfect, 2017; Farha and Brown, 2019; Zhang et al., 2021; Tuci et al., 2025). In recent years, many non-traditional antifungal drugs have demonstrated the potential to inhibit and even kill invasive fungi (Farha and Brown, 2019). Their mechanisms of action are rich and diverse, covering aspects such as interfering with cell wall/membrane synthesis, inhibiting virulence factors, disrupting energy metabolism, and regulating fungal signaling pathways (Lu et al., 2024; Zhen et al., 2024).

Antibacterial agents display notable broad-spectrum antifungal activity. They may be used alone or synergistically with antifungals to alter gene expression related to adhesion, mycelial or biofilm formation, reduce extracellular polysaccharides, decrease surface hydrophobicity, or inhibit efflux pumps. For instance, tobramycin combined with amphotericin B or voriconazole shows synergistic enhancement of *Fusarium* cell wall and membrane permeability (80% and 76% synergy, respectively) (Pozzebon Venturini et al., 2016). Minocycline inhibits *Aspergillus* spp. and *Fusarium* spp. (MIC 0.125–4 μg/mL) and potentiates multiple antifungal drugs (Gao et al., 2020). Polymyxin B binds to anionic membrane lipids (MIC_100_ 8–256 μg/mL) and, when combined with fluconazole, disrupts membranes of *Fusarium*, *Cryptococcus neoformans*, *Rhizopus oryzae*, and *Aspergillus fumigatus*. Animal models confirm antibacterial–antifungal synergy: β-lactams, colistin, and quinolones enhance activity against *Candida* and *Aspergillus* when combined with existing antifungals (Keçeli et al., 2014; Fernández-Rivero et al., 2017; Mohamadzadeh et al., 2020).

Immunosuppressive agents with intrinsic antifungal activity are emerging as candidates for drug repurposing, including calcineurin inhibitors (e.g., cyclosporine, pimecrolimus, tacrolimus/FK506), mTOR inhibitors (e.g., rapamycin), antimetabolites (e.g., mizoribine [MZP], mycophenolic acid [MPA]), and glucocorticoids. Inosine monophosphate dehydrogenase (IMPDH), the rate-limiting enzyme in *de novo* guanine nucleotide biosynthesis, has gained particular attention (Hackstein and Thomson, 2004; Pail et al., 2025; Qin et al., 2025; Tufail et al., 2025). Benzo[b]thiophene-1,1-dioxide, an IMPDH inhibitor, markedly attenuates or abolishes the virulence of emerging *Cryptococcus* isolates and can exhibit fungicidal activity (Kummari et al., 2018). Other IMPDH inhibitors, including MPA and MZP, show potent activity against *Candida albicans* and *Cryptococcus* spp. by disrupting GTP biosynthesis (Kummari et al., 2018). Ribavirin, an antiviral with IMPDH-inhibitory properties, demonstrates *in vitro* and *in vivo* efficacy against *C. albicans*, alone or in combination with azoles, potentially via vacuolar dysfunction and reduced extracellular phospholipase activity (Yousfi et al., 2019; Zhang et al., 2020). Collectively, these data highlight IMPDH as a promising antifungal target, warranting further mechanistic and preclinical evaluation.

Statins were initially known as lipid-lowering and cholesterol-lowering drugs. Statins inhibit HMG-CoA reductase, decreasing ergosterol synthesis and impairing biofilms in *Candida*, *Aspergillus*, and zygomycetes, often synergizing with fluconazole (Macreadie et al., 2006; Callegari et al., 2010; Brilhante et al., 2015). Antiarrhythmics (e.g., verapamil, amiodarone) disrupt calcium homeostasis and efflux pumps, enhancing azole efficacy (da Silva et al., 2013; Yu et al., 2013; Yu et al., 2014; Homa et al., 2017; Alnajjar et al., 2018; Zeng et al., 2019). Antipsychotics (e.g., chlorpromazine, haloperidol derivatives) alter membrane structure or inhibit calmodulin, potentiating antifungal agents (Stylianou et al., 2014; Rossato et al., 2016; Holbrook et al., 2017; Kim et al., 2019). Antidepressants (e.g., fluoxetine, sertraline) damage membranes or inhibit virulence factors, active even against resistant fungi (Gu et al., 2016; Treviño-Rangel et al., 2019; Gowri et al., 2020; Jiang L. et al., 2020). Non-steroidal anti-inflammatory drugs (NSAIDs) such as aspirin and ibuprofen inhibit prostaglandin synthesis, induce reactive oxygen species (ROS) accumulation, and disrupt membrane integrity, leading to fungal death (Ogundeji et al., 2016). Ibuprofen additionally shows anti-spore activity (median MIC 256 μg/mL) and synergizes with amphotericin B, itraconazole, or terbinafine (Borba-Santos et al., 2021). Diclofenac sodium downregulates genes linked to RNA transport and cell cycle in *Aspergillus fumigatus*, reducing mycelial formation (Nargesi and Rezaie, 2018).

Antiparasitic drugs also show antifungal potential. Salicylanilide oxyclozanide has shown activity against *Candida albicans*, including azole- and echinocandin-resistant strains, by disrupting mitochondrial oxidative phosphorylation, collapsing membrane potential, and impairing utilization of non-fermentable carbon sources (Pic et al., 2019). The antimalarial chloroquine, in macrophages infected with *Cryptococcus*, induces fungal death via iron complex formation and inhibits thiamine transporter activity in *Saccharomyces cerevisiae*, linked to glucose metabolism (Huang et al., 2012). In azole-resistant *C. albicans* with abnormal ergosterol synthesis, chloroquine also disrupts morphogenesis (Shinde et al., 2013). Auranofin, an anti-rheumatic drug, inhibits inflammatory pathways and shows broad antifungal activity, including against *Aspergillus fumigatus*, *Apiospora montagnei*, and *Apiospora siamensis*, as well as biofilm inhibition (Thangamani et al., 2016).

Overall, drug repurposing leverages the multi-target potential of existing agents, expands the antifungal arsenal against resistant pathogens, and provides a theoretical foundation for developing combination therapy and novel antifungal strategies (Zhang et al., 2021). However, clinical translation requires thorough pharmacokinetic profiling and large-scale trials to validate efficacy and safety.

### 5.3 Popular target proteins related to fungal resistance

Fungal resistance represents a critical challenge in clinical mycology and antifungal drug development. The identification of resistance-related proteins as therapeutic targets is pivotal for guiding the rational design of antifungal agents. Drug targets are typically macromolecules, such as proteins or nucleic acids, that interact specifically with therapeutic compounds, mediating pharmacological effects or enabling targeted delivery. With the integration of bioinformatics resources, such as complete fungal proteomes from UniProtKB and domain data from Pfam, novel targets can be systematically identified. Furthermore, artificial intelligence (AI) enhances the predictive capacity for small molecule, protein interactions, facilitating the discovery of potent antifungal candidates (Li et al., 2025c; Yin et al., 2025). Resistance mechanisms vary with the mode of action (MOA) of antifungal drugs. In azoles, resistance is commonly attributed to overexpression of efflux pumps (particularly in *Candida*) and alterations in the sterol biosynthesis pathway (Lee et al., 2021). In *Aspergillus fumigatus*, *Cyp51A* point mutations and promoter insertions are major contributors (Garcia-Rubio et al., 2018; Roundtree et al., 2020). In *Cryptococcus neoformans*, chromosomal aneuploidy and hypermutations drive target overexpression and efflux (Iyer et al., 2021; Zhang et al., 2024). Polyenes act by binding ergosterol and disrupting membrane integrity; resistance results from mutations in ergosterol biosynthetic genes. For instance, *Candida albicans* exhibits resistance via *ERG3* deletion and upregulation of *ERG5*, *ERG6*, and *ERG25 (* Yu et al., 2012; Zhou et al., 2018). Transcription factors such as Upc2, which regulate ergosterol biosynthesis, have emerged as critical resistance determinants (Yang et al., 2015; Tan et al., 2022). Heat shock protein Hsp90, involved in stress adaptation, also contributes to antifungal resistance (Wei et al., 2024). Echinocandin resistance is primarily driven by mutations in *FKS1*, while drug-induced cell wall stress can activate tolerance pathways such as the Ca²^+^/calcineurin and Hsp90/mTOR signaling cascades (Perlin, 2007; Walker et al., 2010; Hou et al., 2019; Tian et al., 2024). Pyrimidine analogs like 5-fluorocytosine inhibit nucleic acid synthesis, with resistance arising from *FCY1* mutations (Chang et al., 2021). Additional targets include efflux pumps (e.g., Flu1, Mdr1, Cdr1, Cdr2), kinases and transcription factors (e.g., Snf1, Skr1), signaling proteins (e.g., Hog1, calcineurin), ribosomal proteins (e.g., S3, S6, L4), resistance regulators (e.g., Pdr1), and phosphodiesterases (e.g., Pde1, Pde2) (Day et al., 2018; Usher and Haynes, 2019; Shivarathri et al., 2020; Zgadzay et al., 2022; Kim et al., 2023; Lee et al., 2023; Engle and Kumar, 2024). These proteins offer a theoretical basis for targeted antifungal development. The integration of AI with bioinformatics, *de novo* protein design, and synthetic biology holds promise for precision therapeutics addressing fungal resistance.

### 5.4 Synthetic peptide drugs

Antimicrobial peptides (AMPs) are crucial components of the body’s defense system, exhibiting broad-spectrum antimicrobial activity against various pathogens (Pasupuleti et al., 2012). Peptide-based small-molecule drugs have shown potential in antifungal therapy, such as echinocandins and defensin-derived peptides. These peptide molecules possess several key characteristics, including disrupting cell membrane integrity, inhibiting DNA and protein synthesis, and interfering with cellular metabolic processes and cell wall biosynthesis (Buda De Cesare et al., 2020). Due to their unique mechanism of action, antimicrobial peptides are emerging as potential candidates for the control of drug-resistant fungi. For example, antimicrobial peptides designed based on chromogranin A (CGA) have demonstrated excellent antimicrobial performance. CGA is a protein widely distributed in neurons, and its N-terminal 65–76 amino acid sequence (CGA-N12) has been identified as an antimicrobial peptide with activity (Li et al., 2018; Li X. et al., 2022; Li R. et al., 2023). The uniqueness of CGA-N12 lies in its binding target, KRE9, a highly specific β-1,6-glucan synthase for *Candida albicans*. By inhibiting KRE9 activity, CGA-N12 disrupts the structural integrity of the fungal cell wall, effectively inhibiting fungal growth and reproduction (Li et al., 2018; Li X. et al., 2022). In terms of antifungal activity, CGA-N12 has shown stronger inhibitory effects compared to the traditional antifungal drug fluconazole (Li R. et al., 2023). This difference suggests that CGA-N12, as a novel antimicrobial peptide, has significant potential for future antifungal treatments.

However, peptide small-molecule drugs have various limitations. In terms of effectiveness, there are issues with drug stability and delivery, such as susceptibility to proteases and low oral bioavailability. In terms of target selection and host toxicity, the homology with eukaryotic organisms can interfere with host function, and drug penetration efficiency is insufficient. Additionally, in pharmacokinetics, many drugs have short half-lives, which result in high production costs, strict storage conditions, and poor accessibility. Some drugs also have immunogenicity and allergy risks. Moreover, their antifungal spectrum is narrow, making it difficult to address mixed infections or fungal morphological transitions (Wang et al., 2022; Luo et al., 2023).

Future research directions include structural optimization, new target development, innovations in delivery technologies, and combination therapy strategies. Breakthroughs in these areas require deep interdisciplinary collaboration between structural biology, synthetic chemistry, and clinical needs, balancing efficacy, safety, and accessibility to fill the gaps in antifungal therapy.

### 5.5 Nanotechnology for antifungal drugs

Changing the drug formulation is one of the commonly used and effective methods in drug optimization, significantly improving bioavailability, reducing adverse reactions, and enhancing therapeutic outcomes. For example, RN104 (2- [2-(cyclohexyl methylene) hydrazinyl]-4-phenylthiazole) is a thiazole hydrazone derivative with significant antifungal activity. However, due to its low solubility in physiological pH conditions, the oral bioavailability of RN104 is suboptimal (Silva et al., 2020). To overcome this issue, researchers designed a self-emulsifying drug delivery system (SEDDS) based on RN104 to improve its pharmacokinetic properties and oral bioavailability. In pharmacokinetic studies in mice, RN104-SEDDS significantly increased its oral bioavailability by 2133% compared to free RN104, enhancing its bioactivity (Silva et al., 2024).

Another classic example is AMB, a potent antifungal drug that, when administered intravenously, can cause severe adverse effects, including kidney, liver, and cardiovascular damage, as well as anemia and electrolyte imbalances (Jarvis et al., 2022). To reduce these side effects, a new oral formulation of Amphotericin B, lipid nanocrystal Amphotericin B (MAT2203), has been developed as an alternative to intravenous administration (Gu et al., 2024; Kriegl et al., 2025). The mechanism of action involves targeting cells, such as macrophages, which engulf these lipid nanocrystals and transport them to the infection site. At the infection site, the low intracellular calcium concentration triggers the release mechanism of the nanocrystals, allowing the drug to be directly released inside the cells, thereby avoiding systemic tissue damage caused by Amphotericin B (Boulware et al., 2023). This demonstrates how improvements in drug formulations play a critical role in antifungal drug development, enhancing drug efficacy while significantly reducing adverse effects. Looking ahead, the development of new antifungal drug formulations will undoubtedly bring more possibilities and greater expectations for the treatment of fungal infections.

### 5.6 Antifungal compounds of traditional Chinese medicine will become a new star

**Figure 5:**
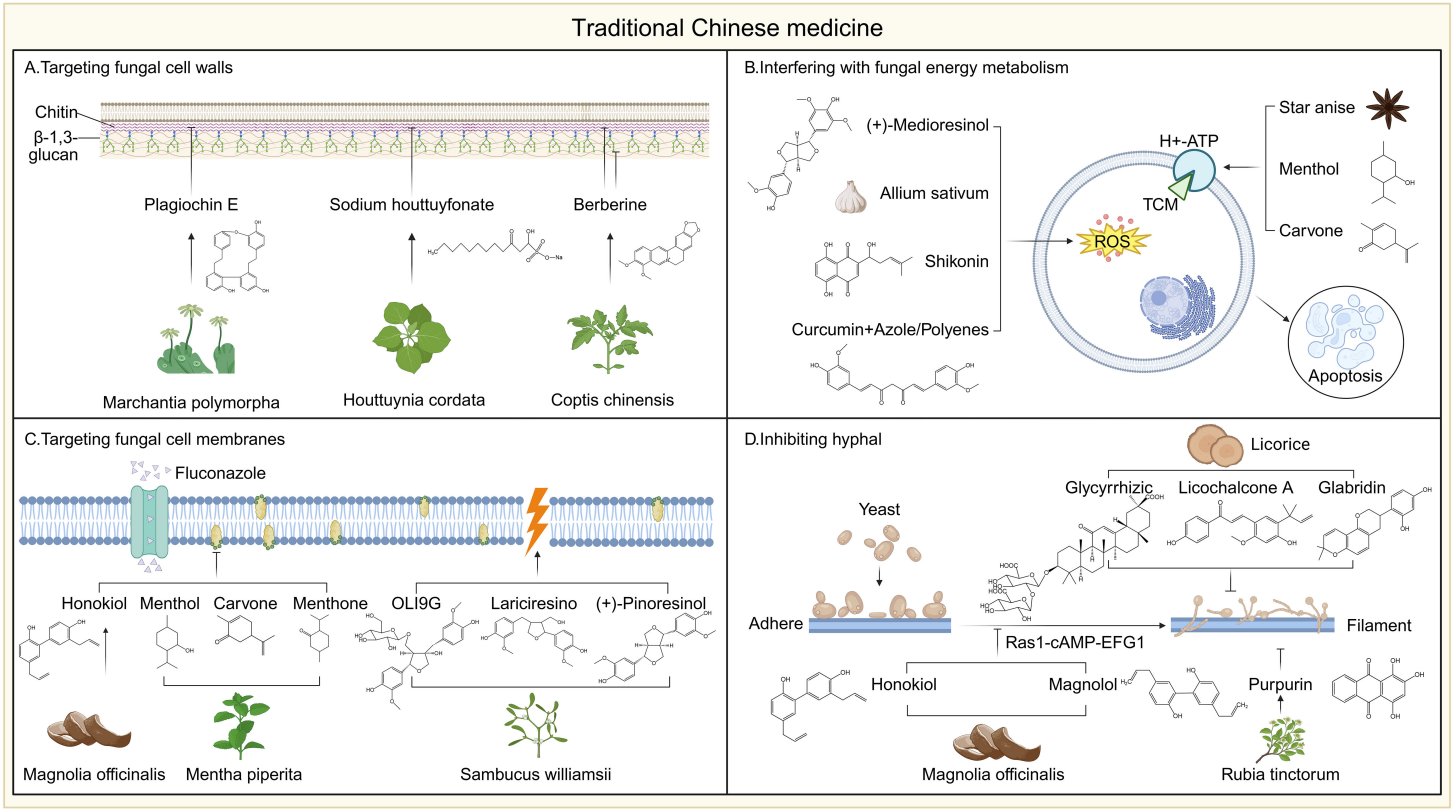
Mechanism of action of traditional Chinese medicine. **(A)** Plagiochin E from *Marchantia polymorpha* inhibited chitin synthesis, Sodium houttuydata from *Houttuynia cordata* regulated gene expression related to β-1,3-glucan synthesis, and berberine from *Coptis chinensis* promoted cell wall components exposure. **(B)** Constituents affecting cell metabolism such as star anise and carvone extracts caused cytosolic acidification by inhibiting H^+^-ATPase, (+)-medioresinol, isolated from *Sambucus williamsii*, *shikonin* and *Allium sativum* (garlic) extracts caused oxidative stress by inducing the accumulation of reactive oxygen species, while curcumin synergistically enhanced the ROS generation ability of other antifungal agents. **(C)** Membrane-targeting components such as honokiol from *Magnolia* and carvone, menthol and menthone from *Mentha piperita* destroy membrane integrity by reducing ergosterol content, while OLI9, lariciresinol, and (+)-pinoresinol from *Sambucus williamsii* directly disrupt the membrane structure. **(D)** Honokiol and magnolol from *Magnolia* blocked hyphal formation by inhibiting the Ras1-cAMP-EFG1 signaling pathway, and licorice-derived compounds such as licochalcone A, glabridin, and glycyrrhizic acid impair fungal growth and morphogenesis. Licochalcone A, Purpurin from *Rubia tinctorum* inhibit fungal morphological development and biofilm formation.

Approximately 80% of antibiotics currently used in clinical practice are derived from natural products, underscoring the value of natural sources in drug discovery (Newman et al., 2003). Traditional Chinese Medicine (TCM), with a long history of treating infectious diseases, has recently gained renewed interest for its potential in combating fungal infections. Increasing evidence suggests that various components of traditional Chinese herbs exhibit significant antifungal activity, functioning through diverse and often complementary mechanisms (Jiang B-C. et al., 2020). TCM-derived compounds have been shown to exert antifungal effects through several pathways, notably by targeting the fungal cell wall and cell membrane, as well as interfering with key metabolic and regulatory processes (**Figure 5**) (Zhang C-W. et al., 2023; Zou et al., 2023).

The fungal cell wall is essential for maintaining cellular integrity and morphology, and its absence in mammalian cells makes it an ideal antifungal target. Several traditional Chinese medicine (TCM)-derived compounds have been shown to disrupt fungal cell wall synthesis or compromise its structural stability. For instance, Plagiochin E, a macrocyclic bis(bibenzyl) compound isolated from *Marchantia polymorpha*, exhibits antifungal activity by targeting chitin synthesis. It significantly downregulates the expression of *CHS1* and alters the expression profile of other chitin synthase genes, leading to impaired chitin biosynthesis and subsequent cell wall damage in *Candida albicans (* Guo et al., 2008; Wu et al., 2008). Sodium houttuyfonate (SH), a sulfur-containing compound derived from *Houttuynia cordata*, has also shown promising effects in combination with fluconazole against *C. albicans*, particularly in resistant strains. SH significantly reduced MIC values when used with fluconazole and exhibited strong synergy (FICI < 0.5). Gene expression analysis revealed that SH modulates the expression of genes involved in β-1,3-glucan synthesis and transport, including upregulation of *ZAP1*, *ADH5*, *XOG1*, and *FKS1*, suggesting its potential mechanism involves enhancing cell wall-targeted antifungal responses (Shao et al., 2017; Liu et al., 2021; Cheng et al., 2023). Berberine, an isoquinoline alkaloid from *Coptis chinensis*, disrupts *Candida albicans* cell-wall architecture by increasing surface exposure of β-glucans and chitin, thereby weakening the barrier and heightening susceptibility to immune attack and antifungal agents (Shi et al., 2017; Liu et al., 2020; Huang et al., 2021). Beyond this remodeling effect, berberine hydrochloride down-regulates the efflux-pump gene *CDR1*, reducing fluconazole extrusion and further sensitizing resistant strains (Zhu et al., 2014). These actions are magnified when berberine is paired with sodium houttuyfonate, which up-regulates β-1,3-glucan synthesis and transport genes, producing marked synergism with fluconazole against recalcitrant *C. albicans (* Tong et al., 2021).

Traditional Chinese medicine (TCM), derived compounds exhibit potent antifungal activity through multiple mechanisms, notably by targeting the fungal cell membrane and its associated components. Ergosterol, a key sterol unique to fungal membranes, is a primary target. For instance, honokiol, a biphenolic compound from *Magnolia officinalis*, has been shown to significantly reduce ergosterol levels in *Candida albicans*, thereby compromising membrane integrity and exerting direct antifungal effects (Sun LM. et al., 2015; Sun et al., 2017). Furthermore, honokiol enhances the efficacy of fluconazole by diminishing the impact of membrane transport proteins, thus reducing drug efflux and increasing intracellular drug accumulation (Jin et al., 2010). Similarly, essential oil components from *Mentha piperita*, including carvone, menthol, and menthone, have been reported to inhibit fungal growth by decreasing ergosterol content in the cell membrane (Samber et al., 2015; Giménez-Santamarina et al., 2022; Hudz et al., 2023). Extracts from *Sambucus williamsii*, such as (−)-olivil-9′-O-β-D-glucopyranoside, lariciresinol, and (+)-pinoresinol, also display membrane-disruptive properties; these compounds depolarize the fungal membrane and increase its permeability, ultimately leading to cell death (Hwang et al., 2010; Hwang et al., 2011; Choi et al., 2013). Beyond direct disruption of membrane lipids, some TCM compounds interfere with membrane-bound proteins and enzymes essential for fungal viability. For example, star anise and peppermint-derived constituents (carvone, menthol, and menthone) inhibit the activity of plasma membrane H^+^-ATPase, causing cytoplasmic acidification and cell death (Edris and Farrag, 2003; Baazeem et al., 2025; Nordin et al., 2025). Additionally, eugenol impairs nutrient uptake by inhibiting amino acid permeases such as Tat1p and Gap1p, further suppressing yeast growth (Darvishi et al., 2013; Nisar et al., 2021). These findings highlight the multifaceted strategies by which TCM compounds compromise fungal membrane function, offering promising avenues for antifungal development, particularly against drug-resistant strains.

TCM components can also exert antifungal effects by inhibiting hyphal and biofilm formation. Several natural compounds have been reported to inhibit both the hyphal morphogenesis and biofilm formation of *Candida albicans* through various mechanisms. For example, two newly identified lignans from *Magnolia*, magnolol and honokiol, suppress the Ras1–cAMP–EFG1 signaling pathway to inhibit hyphal transition (Sun L. et al., 2015). Similarly, licorice-derived compounds such as licochalcone A, glabridin, and glycyrrhizic acid impair fungal growth and morphogenesis (Seleem et al., 2016). Purpurin from *Rubia tinctorum* also demonstrates comparable antifungal activity (Messier and Grenier, 2011; Tsang et al., 2012). In addition, licochalcone A, purpurin, magnolol, and honokiol have been shown to further inhibit biofilm formation (Tsang et al., 2012; Sun L. et al., 2015; Seleem et al., 2016). Given the structural complexity of fungal biofilms, their disruption is particularly valuable. Cinnamaldehyde, derived from cinnamon, significantly impairs biofilm development by *Candida* species (Pires et al., 2011; Khan and Ahmad, 2012). Likewise, berberine, isolated from *Coptis chinensis* and *Hydrastis canadensis*, exhibits notable biofilm-inhibitory effects, especially in combination with miconazole (Wei et al., 2011). The treatment with Paeonol shows effective antifungal and antibiofilm activity against biofilms containing *Candida albicans* and/or *Cryptococcus* spp (Qian et al., 2022). Other natural compounds, including curcumin, thymol, and eugenol, have also been reported to interfere with biofilm formation (Braga et al., 2008; Miladi et al., 2017).

Several Traditional Chinese Medicine (TCM) compounds exert antifungal activity by disrupting fungal energy metabolism, primarily through the induction of oxidative stress and mitochondrial dysfunction. For example, (+)-medioresinol, isolated from *Sambucus williamsii*, promotes the accumulation of reactive oxygen species (ROS) and induces cell cycle arrest, ultimately triggering apoptosis in fungal cells (Hwang et al., 2012). Similarly, baicalin interferes with mitochondrial enzyme activity and cell cycle progression, leading to programmed cell death (Yang et al., 2014). Shikonin has also been shown to increase endogenous ROS levels and impair mitochondrial function, thereby enhancing its antifungal efficacy (Miao et al., 2012). Thymol, a monoterpenoid phenol, suppresses the expression of genes involved in the tricarboxylic acid (TCA) cycle, resulting in diminished energy production and inhibited growth of *Fusarium* species (Zhang et al., 2018). Consistent with these mechanisms, *Allium sativum* (garlic) induces oxidative stress by elevating intracellular ROS levels, further suppressing fungal proliferation (Lemar et al., 2005). Notably, curcumin, a polyphenolic compound derived from turmeric, has been reported to synergize with azoles and polyenes to amplify ROS generation in *Candida albicans*, leading to mitochondrial damage, apoptosis, and enhanced antifungal activity (Sharma et al., 2010b; Sharma et al., 2010a). These findings underscore the diverse mechanisms by which TCM-derived compounds interfere with fungal energy homeostasis and highlight their potential as valuable resources for the development of next-generation antifungal therapeutics.

### 5.7 AI in developing new molecules with antifungal activity

Artificial intelligence (AI) has emerged as a transformative tool in antifungal drug discovery, particularly for identifying and optimizing novel therapeutic agents. By leveraging large-scale biological, chemical, and clinical datasets, AI-driven platforms can accurately predict drug-target interactions, prioritize lead compounds, and optimize molecular structures (Jović and Šmuc, 2020; Zhou et al., 2025). Machine learning (ML) models trained on physicochemical properties of known antifungals effectively distinguish active from inactive compounds, streamlining virtual screening and substantially reducing the candidate space (Das et al., 2021; Randall et al., 2024). Furthermore, ML approaches play a pivotal role in predicting resistance-conferring mutations, while advances such as AlphaFold enable high-precision structural modeling for the identification of antifungal targets (Elfmann and Stülke, 2025; Li et al., 2025c).

The integration of deep generative models and molecular dynamics simulations has further accelerated antifungal discovery. Deep learning, based virtual screening is widely used to predict binding affinities of compounds targeting fungal proteins such as β-(1,3)-D-glucan synthase (Fks1), lanosterol 14α-demethylase (Erg11), and chitin synthase, and other essential genes involved fungal growth. For instance, one study used chemical descriptors to develop a machine learning model targeting *Candida albicans FKS1*, achieving 96.72% classification accuracy (Gao et al., 2022). Another study applied deep learning and molecular docking to screen 1,930 FDA-approved drugs against *C. albicans* dihydrofolate reductase, identifying paritaprevir, lumacaftor, and rifampin as promising inhibitors (Joshi et al., 2022). Addressing the rising incidence of azole-resistant *Aspergillus fumigatus*, researchers also developed novel inhibitors targeting *Af*DHODH, a key enzyme in pyrimidine biosynthesis, yielded 13 candidate molecules, with two demonstrating sub-100 μM activity *in vitro (* Li K. et al., 2025).

AI has also significantly advanced the discovery and design of antifungal peptides (AFPs) (Kavousi et al., 2020; Szymczak et al., 2025). DL-QSARES, a framework integrating deep learning with quantitative structure–activity relationship-based empirical screening, enabled *de novo* design of 49 AFPs, with AFP-13 showing potent activity against *C. albicans* and therapeutic efficacy in animal models (Yin et al., 2025). Other studies using diffusion models and molecular dynamics identified 25 peptides with antifungal properties, among which AMP-29 showed activity against *C. glabrata (* Wang Y. et al., 2025). Advanced AI-platforms like EvoGradient, BroadAMP-GPT, and AMPSphere have demonstrated success in designing antimicrobial peptides with high hit rates against resistant pathogens. EvoGradient used oral microbiome data to generate 32 peptides, all active against at least one ESKAPE pathogen and effective in mouse wound models (Wang B. et al., 2025). BroadAMP-GPT combined AI generation, filtering, and experimental validation, yielding a 57% hit rate against ESKAPE pathogens (Li et al., 2025b). AMPSphere screened over 150,000 genomes to predict 860,000 peptides; among 100 tested, 63 showed antibacterial activity via membrane disruption (Santos-Júnior et al., 2024).

Systems biology has further enhanced AI applications. The integration of comprehensive databases, including genomic, proteomic, and transcriptomic resources, can greatly accelerate the discovery of novel antifungal targets and the identification of genetic variants that confer drug resistance. The first genome-scale metabolic model (GSMM) iRV973 for *Candida auris* predicted growth under various nutrient conditions and identified 50 conserved, serum-essential enzymes—some as novel, non-homologous drug targets (Viana et al., 2023). In *C. albicans*, machine learning and chemogenetic interaction analysis enabled screening of ~6,500 genes, expanding the GRACE (gene replacement and conditional expression) database by 866 entries and revealing 149 fungus-specific essential genes (Fu et al., 2021). This led to the discovery of NP-BTA, a N-pyridinyl-β-thienyl-acrylamide derivative, was discovered to inhibit the essential enzyme glutaminyl-tRNA synthetase (Gln4), representing a novel antifungal mechanism of action (Fu et al., 2021). Recent advances include a predictive machine learning framework utilizing as few as ten genomic variant features to accurately classify isolates as heteroresistant or susceptible, enabling rapid detection of micafungin heteroresistance in *C. parapsilosis (* Zhai et al., 2024). Similarly, an ML-driven multi-omics integration approach, combining transcriptomic and genomic variation data, has effectively pinpointed highly multidrug-adapted traits within the *Cryptococcus gattii* species complex (Fan et al., 2024). Another study used ML on whole-genome annotations and *Erg11* sequences to predict antifungal resistance profiles in yeasts, achieving 75% accuracy for fluconazole resistance and identifying novel resistance-associated residues (Harrison et al., 2025).

The advent of AlphaFold has transformed predictions of antifungal target protein structures and drug–binding interfaces. Leveraging AlphaFold, the structure of Fks1, the β-1,3-glucan synthase targeted by echinocandins, was resolved, revealing resistance-associated mutations, catalytic mechanisms, and the GTP-dependent conformational activation by Rho1 (Hu et al., 2023; Li J. et al., 2025). Furthermore, Simulations of the Erg11–Ncp1 interaction identified residues V234, F235, and L238 in Erg11 as critical for complex stability; disruption of this interface was predicted to sensitize *Candida albicans* to azoles via increased protein misfolding (Li J. et al., 2025). High-throughput deep mutational scanning, informed by AlphaFold, further pinpointed residues in CaErg11 that alter azole binding and resistance (Bédard et al., 2024). Coupling AlphaFold with FoldX identified conserved, non–active site mutations in Fcy1, the 5-fluorocytosine target enzyme, that destabilize the protein, constituting a major mechanism of 5-FC resistance (Després et al., 2022).

In summary, AI technologies, including machine learning, deep learning, and natural language processing—offer powerful solutions to combat antifungal resistance. From drug repurposing and target prediction to peptide design and personalized strategies, AI is poised to drive the next generation of antifungal therapeutics.

## 6 The complex mechanisms behind the failure of fungal treatment

Antifungal treatment failure is a growing clinical challenge, driven not only by pharmacological limitations but also by intrinsic fungal traits. Key factors such as biofilm formation, metabolic flexibility, genetic plasticity, and evolving resistance mechanisms significantly influence therapeutic outcomes (Fisher et al., 2022; Lockhart et al., 2023). Fungi like *Candida* and *Aspergillus* form drug-resistant biofilms, where extracellular matrices limit antifungal penetration and induce dormant “persister” cells (Morelli et al., 2021; Kaur and Nobile, 2023). Under nutrient stress, fungi reprogram metabolism and produce antioxidant enzymes, reducing susceptibility to oxidative agents like AMB (Ke et al., 2024). Additionally, some species, such as *Candida auris*, acquire resistance genes from the environment and modify host conditions to favor survival (Akinbobola et al., 2023). Genetic exchanges and epigenetic modifications further contribute to rapid resistance development (Meng et al., 2024; Zajac et al., 2025; Zhang et al., 2025). Clinical isolates show considerable genomic diversity, including chromosomal duplications and non-classical mutations, complicating treatment strategies (Priest et al., 2022; Rhodes et al., 2022; Ning et al., 2024; Zhou et al., 2024; Brassington et al., 2025). Traditional laboratory strains no longer reflect real-world pathogen behavior, highlighting the need to incorporate contemporary clinical isolates into research. Beyond genetic resistance, fungi exhibit heterogeneous resistance and drug tolerance (Chen et al., 2024; Zhai et al., 2024). Subpopulations may survive high drug concentrations through stress response pathways, while dormant phenotypes persist within host niches like macrophages (Arastehfar et al., 2023). These non-genetic adaptations often precede the emergence of stable, heritable resistance. The rise of multidrug-resistant strains, such as *Candida auris* and *Rhodosporidiobolus fluvialis*, underscores the urgency for new therapeutic approaches (Jackson et al., 2019; Huang et al., 2024). Environmental changes, including climate warming, may drive the emergence of such pathogens by promoting stress adaptation and thermal tolerance (Hofer, 2019; Zhai et al., 2021; Du Toit, 2023; Bottery and Denning, 2024). To address these challenges, antifungal development must shift toward targeting tolerance mechanisms, exploring niche-specific strategies, and expanding surveillance of emerging environmental and clinical pathogens. Future advances will depend on integrating fungal biology with ecological and genomic insights to stay ahead of rapidly adapting fungal threats.

## 7 Conclusion and future outlook

Antifungal drug research is advancing rapidly, driven by the urgent need to improve therapeutic outcomes and combat emerging drug resistance. Current efforts span a wide range of strategies—from structural optimization and target-specific drug design to the development of advanced delivery platforms. A successful response to antifungal resistance demands a multidisciplinary approach that integrates innovative pharmacology, biotechnology, and systems-level understanding of host-pathogen interactions.

One promising avenue lies in the development of antifungal peptide-based therapeutics. Structural optimization through biomimetic engineering, such as modifying host defense peptides or incorporating non-natural amino acids, can enhance stability, membrane permeability, and antifungal activity (Ting et al., 2020). Targeting fungal-specific biological pathways, such as chitin synthesis or virulence factor secretion, offers opportunities for designing highly selective and less toxic agents (Chung et al., 2025). Advances in drug delivery technologies, including liposomes, exosomes, and cell-penetrating peptides (CPPs), further improve bioavailability and tissue targeting (Zhu et al., 2023). Moreover, combination therapies involving immune modulators (e.g., IFN-γ) or antibiofilm agents may synergistically enhance efficacy and delay the onset of resistance (Scriven et al., 2017). The integration of artificial intelligence (AI), synthetic biology, and *de novo* protein design is redefining antifungal drug discovery (Liu et al., 2025). AI-driven computational modeling enables the rational design of antifungal agents and the prediction of resistance mutations, facilitating precision therapeutics. Coupling pathogen biology with host immunology and computational tools can help navigate the complexity of fungal infections and guide the development of targeted interventions. In parallel, technological innovations in pharmaceutical sciences are reshaping antifungal delivery strategies. Nanomaterials, such as mesoporous zinc oxide, are being explored for topical applications, while smart nano-delivery systems targeting the fungal microenvironment represent a promising frontier (Bayat et al., 2022; Thapliyal et al., 2025). These approaches may prolong the efficacy of existing antifungal drugs and accelerate the discovery of new compounds. Improving early diagnostic and monitoring capabilities is equally vital. Rapid detection tools and biomarker-based diagnostic kits can enable timely intervention, bridging prevention and treatment. Emerging data from proteomics and genomic studies suggest that the development of super-resistant fungal strains is associated with critical genomic transitions (Burrack et al., 2022; Lockhart et al., 2023; de Moraes and Ferreira-Pereira, 2024; Joste et al., 2025). Notably, these transitions may involve abrupt conformational changes in key target proteins. Integrating bioinformatics and protein structural biology to monitor such changes could facilitate earlier and more effective interventions.

In summary, the future of antifungal therapy hinges on cross-disciplinary innovation. Emphasis on targeted drug design, advanced delivery platforms, AI-driven prediction models, and early diagnostic tools will be essential in addressing the escalating challenge of antifungal resistance. Moving forward, a proactive approach that couple’s prevention with precision therapy will be critical to shifting the paradigm from treatment to long-term control of fungal diseases.
