# Supplementary material for: New antifungal strategies and drug development against WHO critical priority fungal pathogens
Source: Front Cell Infect Microbiol. 2025 Sep 25;15:1662442. doi: 10.3389/fcimb.2025.1662442 (PMC12507853; doi:10.3389/fcimb.2025.1662442)
Supplement: Supplementary file 1 [file DataSheet1.docx]

| **Trends in Antifungal Resistance among Major Pathogenic Fungi over the Past 25 Years** | | | |
| --- | --- | --- | --- |
| Species | | Region | Overview of drug resistance |
| *Candida* | *C. auris* | Asia | Fluconazole Resistance: 85.87%; Itraconazole Resistance:1.63%; Voriconazole Resistance: 26.09%; Echinocandins Resistance: 0.00%; AMB Resistance: 13.04%. (2009-2017, India) (Chowdhary et al., 2017)  Most *C. auris* isolated from China (98.7%) exhibited resistance to fluconazole, while only a small subset of strains were resistant to amphotericin B (4.2%) and caspofungin (2.2%).(2018-2023, China)(Bing et al., 2024)  The numbers of both clinical cases and geographic areas reporting *C. auris* infections dramatically increased after COVID-19. (2020-2024, China)(Bing et al., 2025) |
|  |  | Europe | Fluconazole Resistance: 88.71%; Itraconazole Resistance: 0.00%; Voriconazole Resistance: 1.61%; Echinocandins Resistance: 0.00%; AMB Resistance: 0.00%. (2009-2017, UK) (Chowdhary et al., 2017)  All 31 tested isolates showed resistance to fluconazole, 5 were resistant to amphotericin B, and they were all susceptible to echinocandins. Crude mortality for infected patients was 68.18%. (2022-2023, Romania)(Surleac et al., 2025)  All *C. auris* isolates (57 isolates) were susceptible to anidulafungin. Of these isolates, 47 (82%) were resistant to fluconazole, 34 (60%) to amphotericin B, four (7%) to caspofungin and three (5%) to micafungin. (2023-2024, Türkiye) (Akkaya et al., 2025) |
|  |  | North America | Fluconazole Resistance: 99.58%; Voriconazole Resistance: 73.59%; Caspofungin Resistance: 0.95%; Micafungin Resistance: 1.06%; Anidulafungin Resistance: 0.95%; Amphotericin B Resistance: 63.52%; Flucytosine Resistance: 1.48%. (2016-2018, US) (Zhu et al., 2020)  Fluconazole Resistance: 92%; amphotericin B Resistance: 32.2%.(2016-2019, Galveston-Houston Gulf Coast Region)(Nguyen and Ren, 2025)  The fluconazole, micafungin, and multidrug resistance rates in C. glabrata that were ~2 times higher than that reported in other regions of the United States. (2019-2021, Minneapolis-Saint Paul region of Minnesota)(Scott et al., 2025) |
|  |  | Latin America | Fluconazole Resistance: 80.00%; Itraconazole Resistance: 0.00%; Voriconazole Resistance: 62.86%; Echinocandins Resistance: 0.00%; AMB Resistance: 31.43%. (2009-2017, isolates from Venezuela and Colombia) (Chowdhary et al., 2017) |
|  |  | Africa | Out of these 15 tested isolates, 10 were resistant to fluconazole, 8 were resistant to amphotericin B, and 5 were resistant to micafungin. Five *C. auris* isolates showed resistance to all three selected antifungals (fluconazole, amphotericin B, and micafungin). (Durban)(Semenya et al., 2025)  *C. auris* was the most common (743 drug resistant strains of 1036 isolates). Forty-five *C. auris* strains were multidrug- and 5, pandrug-resistant.(Ibe and Pohl, 2025) |
|  | *C. glabrata* | Asia | 11.3% (23/204) of the isolates were resistant to fluconazole. (January to December 2021, China)(Xu et al., 2025)  36.84% (7/19) of the isolates were resistant to caspofungin and 15.8% (3/19) of the isolates were resistant to micafungin. (2022-2024, Uttarakhand)(Singh et al., 2025) |
|  |  | Europe | Azole Resistance: 11.00 %; Echinocandin Resistance: 0.50%; Polyene Resistance: 0.0%.(Odoj et al., 2024) (~2013-2022) |
|  |  | North America | 25.6% (21/82) of the isolates were resistant to echinocandins, 29.29% (24/86) of the isolates were resistant to fluconazole. (2008-2015, US)(Misas et al., 2024)  Echinocandin resistance: 2.2–2.7%; Fluconazole resistance: 13.0% (2016-2017)(Castanheira et al., 2020) |
|  |  | Latin America | Echinocandin resistance: 0.0–6.2%; Fluconazole resistance: 0.0% (2016-2017)(Castanheira et al., 2020)  8 % (4/52) resistance rates to fluconazole; 15 % (8/52) resistance rates to micafungin. (2023-2024, Brazil)(Bastos et al., 2025) |
|  |  | Africa | Fluconazole Resistance: 42.1%; Nystatin Resistance: 5.3%; Itraconazole Resistance: 15.8%; Clotrimazole Resistance: 0%. (2020-2023, Volta Region)(Aboagye et al., 2025)  74% (14/19) of the isolates were resistant to zoles. (February and July 2021, Namibia) (Dunaiski et al., 2022) |
|  | *C. parapsilosis* | Asia | A total of 64 (32%) *C. parapsilosis* isolates were non-susceptible to fluconazole, which included resistant (n = 55; MIC >4 mg/L) and susceptible dose-dependent (n = 9) isolates. (2015-2017, India)(Singh et al., 2019)  10.34% (6/58) resistance rate to fluconazole. (2022-2024, Uttarakhand)(Singh et al., 2025)  55.1% (113/205) of the isolates were resistant to zoles fluconazole. (China)(Ning et al., 2024) |
|  |  | Europe | Azole Resistance: 4.00 %; Echinocandin Resistance: 0.00%; Polyene Resistance: 0.0%.(Odoj et al., 2024) (~2013-2022)  Azole resistance: 15.0% (2016-2017)(Castanheira et al., 2020)  All isolates were fluconazole resistant (median minimum inhibitory concentration [MIC] 64 mg/L, minimum 16 to maximum >64 [the highest concentration tested]) and voriconazole resistant (median MIC 1 mg/L, minimum 0·25 to maximum 4), but susceptible to posaconazole. (2016-2022, German) (Brassington et al., 2025)  42.9% (196/457) resistance rate to fluconazole. (2018-2022, Italy and Spain)(Vena et al., 2025) |
|  |  | North America | All *C. parapsilosis* isolates were susceptible to fluconazole, Amphotericin B, Micafungin. (2019-2021, Minneapolis-Saint Paul region of Minnesota)(Scott et al., 2025) |
|  |  | Latin America | 54% (15/28) isolates were fluconazole resistant. (2014-2016, Mexican)(Corzo-Leon et al., 2021) |
|  |  | Africa | 57 (78%) were resistant, 11 (15%) susceptible dose-dependent and 5 (7%) susceptible. (2009-2010, Johannesburg)(Magobo et al., 2020)  464 resistant strains of 622 isolates.(Ibe and Pohl, 2025) |
|  | *C. albicans* | Asia | Fluconazole resistance: 2.94%; Amphotericin B resistance: 14.71% (2022-2024, Uttarakhand)(Singh et al., 2025) |
|  |  | Europe | Azole Resistance: 0.45%; Echinocandin Resistance: 0.00%; Polyene Resistance: 0.0%.(~2013-2022)(Odoj et al., 2024)  Azole Resistance: (0.1%). (2016-2017)(Castanheira et al., 2020) |
|  |  | North America | Azole Resistance: 1.1%. (2016-2017)(Castanheira et al., 2020) |
|  |  | Latin America | Azole Resistance: 1.0%. (2016-2017)(Castanheira et al., 2020)  6 % resistance rates to fluconazole. (2023-2024, Brazil)(Bastos et al., 2025) |
|  |  | Africa | *C. albicans* were 606 isolates out of which 23 strains were resistant.(Ibe and Pohl, 2025)  Fluconazole Resistance: 53.2%; Nystatin Resistance: 12.8%; Itraconazole Resistance: 53.2%; Clotrimazole Resistance: 2.1%. (2020-2023, Volta Region)(Aboagye et al., 2025)  2% (2/90) of the isolates were resistant to zoles. (February and July 2021, Namibia)(Dunaiski et al., 2022) |
| *Cryptococcus* | *C. neoformans* | Asia | A total of 86 of the isolates (43.2%) were not sensitive to fluconazole at a MIC_50_ ≥ 8 μg/ml, most of the isolates were resistant to amphotericin B, and nearly all isolates were resistant to itraconazole and posaconazole. Resistances to 5-Flucytosine and voriconazole were very rare. (2015-2020, China) (Yang et al., 2021a)  All 86 cryptococcal isolates were susceptible to 5-Flucytocine, Amphotericin B, Fluconazole, Itriconazole, and Voriconazole. (2016-2017, China)(Chen et al., 2018)  The epidemiological cutoff values for itraconazole (7; 50 % of the isolates) and for posaconazole (1; 7.1 % of the isolate), were one log2 dilution higher than the wild type range. (2016-2019, Iran)(Bandalizadeh et al., 2020)  High antifungal minimal inhibitory concentrations (MICs) above the epidemiological cutoff values (ECVs) were observed in isolates, and more than half of isolates were non-wild-type to amphotericin B (89.15%, 109/133). Eight isolates were resistant to fluconazole, and eight isolates were non-wild type to 5-fluorocytosine. (2017-2020, China)(Zhou et al., 2022)  The percentages of non-wild type to amphotericin B, 5-fluorocytosine, fluconazole, itraconazole, voriconazole, and posaconazole were 10.0 %, 4.0 %, 8.0 %, 6.0 %, 2.0 %, and 8.0 %, respectively. (2020-2023, Korea)(Park et al., 2025)  Two clinical *C. neoformans var. grubii* isolates that were resistant to 5-flucytosine (MIC >64 µg ml⁻¹). The environmental *C. neoformans* *var. grubii* isolates were significantly less susceptible to fluconazole, itraconazole and 5-flucytosine (P<0.0001) than the clinical isolates. (Western India)(Chowdhary et al., 2011) |
|  |  | Europe | The mean prevalence of fluconazole resistance was 12.1% (95% confidence interval [CI]: 6.7-17.6) for all isolates (n = 4995). Mean fluconazole resistance was 10.6% (95% CI: 5.5-15.6) for the incident isolates (n = 4747) and 24.1% (95% CI: -3.1-51.2) for the relapse isolates (n = 248). Of the 4995 isolates, 936 (18.7%) had MICs above the ecological cut-off value. (1988-2017, UK)(Bongomin et al., 2018)  All isolates were amphotericin B susceptible. Flucytosine MIC was elevated for 1/61 isolates (>32 mg l (-1)). (Denmark)(Hagen et al., 2016) |
|  |  | North America | *C. neoformans* complex isolates with a fluconazole MIC  8 mg/L, 6 (46%) were observed in the most recent 5-year period. (2000-2019, US)(Stewart et al., 2025)  Within *C. neoformans*, genotype VNII had significantly lower geometric mean MICs for fluconazole than genotype VNI. (US)(Chong et al., 2010) |
|  |  | Latin America | Susceptibility patterns before treatment to AMB showed MICs < or =1 microg/ml for all the strains, and no increase was seen after treatment. All the strains were susceptible to FCZ (MIC< or =8 microg/ml) at diagnosis. (1999-2005, Argentina)(Arechavala et al., 2009)  From the 570 isolates, all have data on antifungal susceptibility to fluconazole, 434 to amphotericin-B, 296 to itraconazole, 251 to voriconazole, 250 to 5-fluorocytosine, and 192 to posaconazole. (1999-2017)(Firacative et al., 2021)  Antifungal susceptibility testing revealed that most isolates fell below the local epidemiological cut-off value. (2008-2013, 2017-2021, Uruguay)(Puime et al., 2023)  Non-wild-type phenotype to the antifungals was observed in 26.4% (19/72) of the *C. neoformans* and *C. deuterogattii* clinical isolates, and the latter demonstrated higher MIC to fluconazole and itraconazole than *C. neoformans* clinical isolates. (2012-2017, south-eastern Brazil)(Grizante Barião et al., 2020)  *C. neoformans* VNI modal MIC of voriconazole is 0.06 μg/ml; MIC of fluconazole is 4 μg/ml; MICs for amphotericin B were 1 μg/ml. (Brazilian Amazon Region)(Nishikawa et al., 2019) |
|  |  | Africa | The fluconazole MIC50, MIC90 and geometric mean MIC was 4 μg/ml, 8 μg/ml and 4.11 μg/ml in 2017 (n = 229) compared to 1 μg/ml, 2 μg/ml and 2.08 μg/ml in 2007-2008 (n = 249) respectively. Voriconazole, itraconazole and posaconazole Etests were performed on 16 of 229 (7%) *C. neoformans* isolates with a fluconazole MIC value of ≥16 μg/ml; only one had MIC values of >32 μg/ml for these three antifungal agents. (1 January through 31 March 2017, South African)(Naicker et al., 2020)  *C. neoformans* isolates showed higher minimum inhibitory concentrations (MICs) to FCZ, ITZ, and voriconazole (VRZ) than those of *C. gattii* VGI and VGII. (Egypt)(Taha et al., 2024) |
|  |  | Oceania | All isolates demonstrated low MICs to antifungal agents including fluconazole. Similarly fluconazole geometric mean MICs against fluconazole for VNI (2.3 mg/l) were significantly higher than VNII (0.87 mg/l, P = .036). Geometric mean MIC values against 5-fluorocytosine for VNI (1.741 mg/l) were significantly higher than those for VGI (0.47 mg/l, P = .002).(Lee et al., 2019) |
|  | *C. gattii* | Asia | Antifungal susceptibility testing showed a low MIC90 (0.25 μg ml (-1)) of the new azoles posaconazole and isavuconazole for these environmental isolates. (India)(Chowdhary et al., 2013)  The MIC₉₀ of *C. gattii* was twofold higher than that of *C. neoformans var. grubii* for fluconazole, itraconazole and voriconazole. (Western India)(Chowdhary et al., 2011) |
|  |  | Europe | All isolates were amphotericin B susceptible. Isolates were somewhat less susceptible to the azoles. MICs of fluconazole (>32 mg l (-1)), voriconazole (≥0.5 mg l (-1)) and isavuconazole (0.06 and 0.25 mg l (-1) respectively) were elevated compared to the wild-type population. (Denmark)(Hagen et al., 2016) |
|  |  | North America | *C. gattii* isolates belonging to molecular genotype VGII had significantly higher MIC values for flucytosine and all azole antifungal agents tested, particularly fluconazole, than isolates of other *C. gattii* genotypes. VGII isolates from the north and west of Australia required higher drug levels for inhibition than those from Vancouver Island, Canada. (US)(Chong et al., 2010)  23.3% of the Pacific Northwest isolates have fluconazole MICs of 16 to 32 μg/ml is roughly double the 12.7% of isolates in this category reported from Spain. (US)(Morera-López et al., 2005; Iqbal et al., 2010) |
|  |  | Latin America | From the 570 isolates, all have data on antifungal susceptibility to fluconazole, 434 to amphotericin-B, 296 to itraconazole, 251 to voriconazole, 250 to 5-fluorocytosine, and 192 to posaconazole. (1999-2017)(Firacative et al., 2021)  Antifungal susceptibility tests showed that itraconazole, voriconazole and posaconazole had high activity against all isolates, while amphotericin B, fluconazole and 5-fluorocytosine were the least active drugs against all studied isolates. (2003-2019, Argentina)(Taverna et al., 2023)  Antifungal susceptibility testing revealed that most isolates fell below the local epidemiological cut-off value. (2008-2013, 2017-2021, Uruguay)(Puime et al., 2023)  Non-wild-type phenotype to the antifungals was observed in 26.4% (19/72) of the *C. neoformans* and *C. deuterogattii* clinical isolates, and the latter demonstrated higher MIC to fluconazole and itraconazole than C. neoformans clinical isolates. (2012-2017, south-eastern Brazil)(Grizante Barião et al., 2020)  *C. gattii* VGII modal MIC of voriconazole is 0.25 μg/ml; MIC of fluconazole is 4 μg/ml; MICs for amphotericin B were 1 μg/ml. (Brazilian Amazon Region)(Nishikawa et al., 2019) |
|  |  | Africa | *C. gattii* VGIII was less susceptible to fluconazole (FCZ) and itraconazole (ITZ) than VGI and VGII. (Egypt)(Taha et al., 2024) |
|  |  | Oceania | All isolates demonstrated low MICs to antifungal agents including fluconazole. Geometric mean MIC values against 5-fluorocytosine for VNI (1.741 mg/l) were significantly higher than those for VGI (0.47 mg/l, P = .002).(Lee et al., 2019) |
| *Aspergillus* | *A. fumigatus* | Asia | Itraconazole resistance: 0.9%; Voriconazole resistance:0.7%; Itraconazole and Posaconazole resistance: 1.3%; Itraconazole and Voriconazole and Posaconazole resistance: 1.3%. (1999-2019, China)(Yang et al., 2021b)  Azole resistance: 6%. (Asia-Western Pacific region, 2011-2019)(Pfaller et al., 2021)  Azole resistance: 1.20%. (2023-2024, China)(Kang et al., 2025)  Overall drug resistance:8.29%. (~2024)(Tashiro et al., 2025)  The pooled proportion of triazole-resistant *A. fumigatus* (TRAF) in clinical isolates (predominantly respiratory > ear > other sources) was 4%. (~2025)(Swain et al., 2025) |
|  |  | Europe | Dutch nationwide surveillance shows triazole resistance rose from <1% to ~4–7% (1996–2001: 0.79%; 2002–2006: 4.25%). (Netherlands)(Buil et al., 2019)  106 (49%) showed resistance to at least 1 of the tested antifungal drugs. (2005-2017, England, Wales, Scotland and Ireland)(Rhodes et al., 2022)  Resistance frequency increased from 7.6% in 2013 (58/760 patients) to 14.7% in 2018.The mean voriconazole MIC of TR_34_/L98H isolates decreased from 8 mg/L (2013) to 2 mg/L (2018), and the voriconazole-resistance frequency was 34% lower in 2018 than in 2013. (2013-2018, Netherlands)(Lestrade et al., 2020)  Over the 5-year period 16 (11.3%) of 142 *A. fumigatus* culture-positive animals harbored an azole-resistant isolate. (2015-2020, Netherlands)(van Dijk et al., 2024)  Overall drug resistance:7.03%. (~2024)(Tashiro et al., 2025) |
|  |  | North America | Clade 1 azole resistance: 36.9% (171/463). Clade 2 azole resistance: 74.0% (154/208). Clade 3 azole resistance: 51.2% (20/39); pan-azole resistance: 5.12% (2/39). (2018-2019, US)(Celia-Sanchez et al., 2024)  Overall drug resistance:3.4%. (~2024)(Tashiro et al., 2025) |
|  |  | Latin America | Overall drug resistance:7.77%. (~2024)(Tashiro et al., 2025) |
|  |  | Africa | All isolates were susceptible to the tested antifungals. (2021-2022, Nigeria)(Davies et al., 2025)  Overall drug resistance:3.37%. (~2024)(Tashiro et al., 2025) |
|  |  | Oceania | Overall drug resistance:2.75%. (~2024)(Tashiro et al., 2025) |

**Reference**:

Aboagye, G., Waikhom, S., Asiamah, E.A., Tettey, C.O., Mbroh, H., Smith, C., et al. (2025). Antifungal susceptibility profiles of Candida and non-albicans species isolated from pregnant women: implications for emerging antimicrobial resistance in maternal health. *Microbiol Spectr* 13, e0078725. doi: 10.1128/spectrum.00787-25.

Akkaya, Y., Erdin, B.N., Yılmaz, A.M., Kılıç İ, H., and Toraman, Z.A. (2025). Identification and antifungal resistance profiling of Candida (Candidozyma) auris in a tertiary hospital in Istanbul, Türkiye. *Ann Saudi Med* 45, 207-217. doi: 10.5144/0256-4947.2025.207.

Arechavala, A.I., Ochiuzzi, M.E., Borgnia, M.D., and Santiso, G.M. (2009). Fluconazole and amphotericin B susceptibility testing of Cryptococcus neoformans: results of minimal inhibitory concentrations against 265 isolates from HIV-positive patients before and after two or more months of antifungal therapy. *Rev Iberoam Micol* 26, 194-197. doi: 10.1016/j.riam.2009.02.001.

Bandalizadeh, Z., Shokohi, T., Badali, H., Abastabar, M., Babamahmoudi, F., Davoodi, L., et al. (2020). Molecular epidemiology and antifungal susceptibility profiles of clinical Cryptococcus neoformans/Cryptococcus gattii species complex. *J Med Microbiol* 69, 72-81. doi: 10.1099/jmm.0.001101.

Bastos, R.W., de Aguiar Peres, N.T., da Silva, K.J.G., Eufrasio, L.G., de Carvalho, D.S., Cruz, G.S., et al. (2025). Antifungal resistance in yeasts from One Health perspective: A Brazilian study. *Sci Total Environ* 973, 179139. doi: 10.1016/j.scitotenv.2025.179139.

Bing, J., Du, H., Guo, P., Hu, T., Xiao, M., Lu, S., et al. (2024). Candida auris-associated hospitalizations and outbreaks, China, 2018-2023. *Emerg Microbes Infect* 13, 2302843. doi: 10.1080/22221751.2024.2302843.

Bing, J., Huang, Y., Du, H., Guo, P., Cao, J., Kang, M., et al. (2025). Rapid spread of Candida auris in China after COVID-19. *J Infect* 90, 106476. doi: 10.1016/j.jinf.2025.106476.

Bongomin, F., Oladele, R.O., Gago, S., Moore, C.B., and Richardson, M.D. (2018). A systematic review of fluconazole resistance in clinical isolates of Cryptococcus species. *Mycoses* 61, 290-297. doi: 10.1111/myc.12747.

Brassington, P.J.T., Klefisch, F.R., Graf, B., Pfüller, R., Kurzai, O., Walther, G., et al. (2025). Genomic reconstruction of an azole-resistant Candida parapsilosis outbreak and the creation of a multi-locus sequence typing scheme: a retrospective observational and genomic epidemiology study. *Lancet Microbe* 6, 100949. doi: 10.1016/j.lanmic.2024.07.012.

Buil, J.B., Snelders, E., Denardi, L.B., Melchers, W.J.G., and Verweij, P.E. (2019). Trends in Azole Resistance in Aspergillus fumigatus, the Netherlands, 1994-2016. *Emerg Infect Dis* 25, 176-178. doi: 10.3201/eid2501.171925.

Castanheira, M., Deshpande, L.M., Messer, S.A., Rhomberg, P.R., and Pfaller, M.A. (2020). Analysis of global antifungal surveillance results reveals predominance of Erg11 Y132F alteration among azole-resistant Candida parapsilosis and Candida tropicalis and country-specific isolate dissemination. *Int J Antimicrob Agents* 55, 105799. doi: 10.1016/j.ijantimicag.2019.09.003.

Celia-Sanchez, B.N., Mangum, B., Gómez Londoño, L.F., Wang, C., Shuman, B., Brewer, M.T., et al. (2024). Pan-azole- and multi-fungicide-resistant Aspergillus fumigatus is widespread in the United States. *Appl Environ Microbiol* 90, e0178223. doi: 10.1128/aem.01782-23.

Chen, Y.H., Yu, F., Bian, Z.Y., Hong, J.M., Zhang, N., Zhong, Q.S., et al. (2018). Multilocus Sequence Typing Reveals both Shared and Unique Genotypes of Cryptococcus neoformans in Jiangxi Province, China. *Sci Rep* 8, 1495. doi: 10.1038/s41598-018-20054-4.

Chong, H.S., Dagg, R., Malik, R., Chen, S., and Carter, D. (2010). In vitro susceptibility of the yeast pathogen cryptococcus to fluconazole and other azoles varies with molecular genotype. *J Clin Microbiol* 48, 4115-4120. doi: 10.1128/jcm.01271-10.

Chowdhary, A., Prakash, A., Randhawa, H.S., Kathuria, S., Hagen, F., Klaassen, C.H., et al. (2013). First environmental isolation of Cryptococcus gattii, genotype AFLP5, from India and a global review. *Mycoses* 56, 222-228. doi: 10.1111/myc.12039.

Chowdhary, A., Randhawa, H.S., Sundar, G., Kathuria, S., Prakash, A., Khan, Z., et al. (2011). In vitro antifungal susceptibility profiles and genotypes of 308 clinical and environmental isolates of Cryptococcus neoformans var. grubii and Cryptococcus gattii serotype B from north-western India. *J Med Microbiol* 60, 961-967. doi: 10.1099/jmm.0.029025-0.

Chowdhary, A., Sharma, C., and Meis, J.F. (2017). Candida auris: A rapidly emerging cause of hospital-acquired multidrug-resistant fungal infections globally. *PLoS Pathog* 13, e1006290. doi: 10.1371/journal.ppat.1006290.

Corzo-Leon, D.E., Peacock, M., Rodriguez-Zulueta, P., Salazar-Tamayo, G.J., and MacCallum, D.M. (2021). General hospital outbreak of invasive candidiasis due to azole-resistant Candida parapsilosis associated with an Erg11 Y132F mutation. *Med Mycol* 59, 664-671. doi: 10.1093/mmy/myaa098.

Davies, A.A., Spruijtenburg, B., Meijer, E.F.J., Osaigbovo, II, Balogun, O., Adekoya, A., et al. (2025). Molecular identification and antifungal susceptibility testing of Aspergillus species among patients with chronic pulmonary aspergillosis in Nigeria. *Afr J Lab Med* 14, 2674. doi: 10.4102/ajlm.v14i1.2674.

Dunaiski, C.M., Kock, M.M., Jung, H., and Peters, R.P.H. (2022). Importance of Candida infection and fluconazole resistance in women with vaginal discharge syndrome in Namibia. *Antimicrob Resist Infect Control* 11, 104. doi: 10.1186/s13756-022-01143-6.

Firacative, C., Meyer, W., and Castañeda, E. (2021). Cryptococcus neoformans and Cryptococcus gattii Species Complexes in Latin America: A Map of Molecular Types, Genotypic Diversity, and Antifungal Susceptibility as Reported by the Latin American Cryptococcal Study Group. *J Fungi (Basel)* 7. doi: 10.3390/jof7040282.

Grizante Barião, P.H., Tonani, L., Cocio, T.A., Martinez, R., Nascimento, É., and von Zeska Kress, M.R. (2020). Molecular typing, in vitro susceptibility and virulence of Cryptococcus neoformans/Cryptococcus gattii species complex clinical isolates from south-eastern Brazil. *Mycoses* 63, 1341-1351. doi: 10.1111/myc.13174.

Hagen, F., Hare Jensen, R., Meis, J.F., and Arendrup, M.C. (2016). Molecular epidemiology and in vitro antifungal susceptibility testing of 108 clinical Cryptococcus neoformans sensu lato and Cryptococcus gattii sensu lato isolates from Denmark. *Mycoses* 59, 576-584. doi: 10.1111/myc.12507.

Ibe, C., and Pohl, C.H. (2025). Epidemiology and drug resistance among Candida pathogens in Africa: Candida auris could now be leading the pack. *Lancet Microbe* 6, 100996. doi: 10.1016/j.lanmic.2024.100996.

Iqbal, N., DeBess, E.E., Wohrle, R., Sun, B., Nett, R.J., Ahlquist, A.M., et al. (2010). Correlation of genotype and in vitro susceptibilities of Cryptococcus gattii strains from the Pacific Northwest of the United States. *J Clin Microbiol* 48, 539-544. doi: 10.1128/jcm.01505-09.

Kang, Y., Ma, W., Li, Q., Wang, P., and Jia, W. (2025). Epidemiology, antifungal susceptibility and biological characteristics of clinical Aspergillus fumigatus in a tertiary hospital. *Sci Rep* 15, 16906. doi: 10.1038/s41598-025-00187-z.

Lee, G.A., Arthur, I., Merritt, A., and Leung, M. (2019). Molecular types of Cryptococcus neoformans and Cryptococcus gattii in Western Australia and correlation with antifungal susceptibility. *Med Mycol* 57, 1004-1010. doi: 10.1093/mmy/myy161.

Lestrade, P.P.A., Buil, J.B., van der Beek, M.T., Kuijper, E.J., van Dijk, K., Kampinga, G.A., et al. (2020). Paradoxal Trends in Azole-Resistant Aspergillus fumigatus in a National Multicenter Surveillance Program, the Netherlands, 2013-2018. *Emerg Infect Dis* 26, 1447-1455. doi: 10.3201/eid2607.200088.

Magobo, R.E., Lockhart, S.R., and Govender, N.P. (2020). Fluconazole-resistant Candida parapsilosis strains with a Y132F substitution in the ERG11 gene causing invasive infections in a neonatal unit, South Africa. *Mycoses* 63, 471-477. doi: 10.1111/myc.13070.

Misas, E., Seagle, E., Jenkins, E.N., Rajeev, M., Hurst, S., Nunnally, N.S., et al. (2024). Genomic description of acquired fluconazole- and echinocandin-resistance in patients with serial Candida glabrata isolates. *J Clin Microbiol* 62, e0114023. doi: 10.1128/jcm.01140-23.

Morera-López, Y., Torres-Rodríguez, J.M., Jiménez-Cabello, T., and Baró-Tomás, T. (2005). Cryptococcus gattii: in vitro susceptibility to the new antifungal albaconazole versus fluconazole and voriconazole. *Med Mycol* 43, 505-510. doi: 10.1080/13693780400029528.

Naicker, S.D., Mpembe, R.S., Maphanga, T.G., Zulu, T.G., Desanto, D., Wadula, J., et al. (2020). Decreasing fluconazole susceptibility of clinical South African Cryptococcus neoformans isolates over a decade. *PLoS Negl Trop Dis* 14, e0008137. doi: 10.1371/journal.pntd.0008137.

Nguyen, M.D., and Ren, P. (2025). Trends in Antifungal Resistance Among Candida Species: An Eight-Year Retrospective Study in the Galveston-Houston Gulf Coast Region. *J Fungi (Basel)* 11. doi: 10.3390/jof11030232.

Ning, Y., Dai, R., Zhang, L., Xu, Y., and Xiao, M. (2024). Copy number variants of ERG11: mechanism of azole resistance in Candida parapsilosis. *Lancet Microbe* 5, e10. doi: 10.1016/s2666-5247(23)00294-x.

Nishikawa, M.M., Almeida-Paes, R., Brito-Santos, F., Nascimento, C.R., Fialho, M.M., Trilles, L., et al. (2019). Comparative antifungal susceptibility analyses of Cryptococcus neoformans VNI and Cryptococcus gattii VGII from the Brazilian Amazon Region by the Etest, Vitek 2, and the Clinical and Laboratory Standards Institute broth microdilution methods. *Med Mycol* 57, 864-873. doi: 10.1093/mmy/myy150.

Odoj, K., Garlasco, J., Pezzani, M.D., Magnabosco, C., Ortiz, D., Manco, F., et al. (2024). Tracking Candidemia Trends and Antifungal Resistance Patterns across Europe: An In-Depth Analysis of Surveillance Systems and Surveillance Studies. *J Fungi (Basel)* 10. doi: 10.3390/jof10100685.

Park, B., Suh, E., Choi, Y., Kim, T.Y., Won, E.J., Huh, H.J., et al. (2025). Microbiological and clinical epidemiology of cryptococcosis in non-HIV Korean patients: 4-year collection. *J Infect Public Health* 18, 102842. doi: 10.1016/j.jiph.2025.102842.

Pfaller, M.A., Carvalhaes, C.G., Rhomberg, P., Messer, S.A., and Castanheira, M. (2021). Antifungal susceptibilities of opportunistic filamentous fungal pathogens from the Asia and Western Pacific Region: data from the SENTRY Antifungal Surveillance Program (2011-2019). *J Antibiot (Tokyo)* 74, 519-527. doi: 10.1038/s41429-021-00431-4.

Puime, C.A., Bórmida, V., and Pan, D. (2023). Molecular typing and antifungal susceptibility of clinical isolates of Cryptococcus neoformans and Cryptococcus gattii species complexes from the National Invasive Fungal Surveillance Network of Uruguay. *Med Mycol* 61. doi: 10.1093/mmy/myad059.

Rhodes, J., Abdolrasouli, A., Dunne, K., Sewell, T.R., Zhang, Y., Ballard, E., et al. (2022). Population genomics confirms acquisition of drug-resistant Aspergillus fumigatus infection by humans from the environment. *Nat Microbiol* 7, 663-674. doi: 10.1038/s41564-022-01091-2.

Scott, N.E., Wash, E., Zajac, C., Erayil, S.E., Kline, S.E., and Selmecki, A. (2025). Heterogeneity of Candida bloodstream isolates in an academic medical center and affiliated hospitals. *Microbiol Spectr* 13, e0046425. doi: 10.1128/spectrum.00464-25.

Semenya, M.D., Aladejana, A.E., and Ndlovu, S.I. (2025). Characterization of susceptibility patterns and adaptability of the newly emerged Candida auris. *Int Microbiol* 28, 575-587. doi: 10.1007/s10123-024-00563-1.

Singh, A., Singh, P.K., de Groot, T., Kumar, A., Mathur, P., Tarai, B., et al. (2019). Emergence of clonal fluconazole-resistant Candida parapsilosis clinical isolates in a multicentre laboratory-based surveillance study in India. *J Antimicrob Chemother* 74, 1260-1268. doi: 10.1093/jac/dkz029.

Singh, R., Kakati, B., and Mittal, G. (2025). Trends and Characteristics of Candidemia in Patients With Suspected Sepsis: A Two-Year Retrospective Study From a Tertiary Hospital in Uttarakhand. *Cureus* 17, e86241. doi: 10.7759/cureus.86241.

Stewart, A.G., Laupland, K.B., Edwards, F., Gassiep, I., Koo, S., Hammond, S.P., et al. (2025). Clinical Characteristics and Outcomes in Patients With Cryptococcaemia From a Large Population-Based Cohort. *Mycoses* 68, e70091. doi: 10.1111/myc.70091.

Surleac, M., Stanciu, A.M., Florea, D., Paraschiv, S., Tălăpan, D., Flonta, M., et al. (2025). A clinical and molecular analysis of Candidozyma auris strains from Romania, 2022-2023. *Microbiol Spectr* 13, e0280924. doi: 10.1128/spectrum.02809-24.

Swain, S., Ajayababu, A., Chowdhury, S., Singh, G., and Ray, A. (2025). Epidemiology of Triazole Resistant Aspergillus fumigatus in Asia: A Systematic Review and Meta-Analysis. *Mycoses* 68, e70099. doi: 10.1111/myc.70099.

Taha, M., Tartor, Y.H., Elaziz, R.M.A., and Elsohaby, I. (2024). Genetic diversity and antifungal susceptibilities of environmental Cryptococcus neoformans and Cryptococcus gattii species complexes. *IMA Fungus* 15, 21. doi: 10.1186/s43008-024-00153-w.

Tashiro, M., Nakano, Y., Shirahige, T., Kakiuchi, S., Fujita, A., Tanaka, T., et al. (2025). Comprehensive Review of Environmental Surveillance for Azole-Resistant Aspergillus fumigatus: A Practical Roadmap for Hospital Clinicians and Infection Control Teams. *J Fungi (Basel)* 11. doi: 10.3390/jof11020096.

Taverna, C.G., Arias, B.A., Firacative, C., Vivot, M.E., Szusz, W., Vivot, W., et al. (2023). Genotypic Diversity and Antifungal Susceptibility of Clinical Isolates of Cryptococcus Gattii Species Complex from Argentina. *Mycopathologia* 188, 51-61. doi: 10.1007/s11046-022-00705-x.

van Dijk, M.A.M., Buil, J.B., Tehupeiory-Kooreman, M., Broekhuizen, M.J., Broens, E.M., Wagenaar, J.A., et al. (2024). Azole Resistance in Veterinary Clinical Aspergillus fumigatus Isolates in the Netherlands. *Mycopathologia* 189, 50. doi: 10.1007/s11046-024-00850-5.

Vena, A., Tiseo, G., Falcone, M., Bartalucci, C., Marelli, C., Cesaretti, M., et al. (2025). Impact of Fluconazole Resistance on the Outcomes of Patients With Candida parapsilosis Bloodstream Infections: A Retrospective Multicenter Study. *Clin Infect Dis* 80, 540-550. doi: 10.1093/cid/ciae603.

Xu, X., Sun, Y., Hu, D., Tsui, C.K., Zhang, L., and Deng, S. (2025). Molecular epidemiology of Nakaseomyces glabrata associated with vulvovaginal candidiasis revealed high genetic variability and the presence of novel genotypes in China. *Virulence* 16, 2543058. doi: 10.1080/21505594.2025.2543058.

Yang, C., Bian, Z., Blechert, O., Deng, F., Chen, H., Li, Y., et al. (2021a). High Prevalence of HIV-Related Cryptococcosis and Increased Resistance to Fluconazole of the Cryptococcus neoformans Complex in Jiangxi Province, South Central China. *Front Cell Infect Microbiol* 11, 723251. doi: 10.3389/fcimb.2021.723251.

Yang, X., Chen, W., Liang, T., Tan, J., Liu, W., Sun, Y., et al. (2021b). A 20-Year Antifungal Susceptibility Surveillance (From 1999 to 2019) for Aspergillus spp. and Proposed Epidemiological Cutoff Values for Aspergillus fumigatus and Aspergillus flavus: A Study in a Tertiary Hospital in China. *Front Microbiol* 12, 680884. doi: 10.3389/fmicb.2021.680884.

Zhou, Z., Zhu, C., Ip, M., Liu, M., Zhu, Z., Liu, R., et al. (2022). Molecular Epidemiology and Antifungal Resistance of Cryptococcus neoformans From Human Immunodeficiency Virus-Negative and Human Immunodeficiency Virus-Positive Patients in Eastern China. *Front Microbiol* 13, 942940. doi: 10.3389/fmicb.2022.942940.

Zhu, Y., O'Brien, B., Leach, L., Clarke, A., Bates, M., Adams, E., et al. (2020). Laboratory Analysis of an Outbreak of Candida auris in New York from 2016 to 2018: Impact and Lessons Learned. *J Clin Microbiol* 58. doi: 10.1128/jcm.01503-19.
